# Response to Waterlogging Stress in Wild and Domesticated Accessions of Timothy (*Phleum pratense*) and Its Relatives *P. alpinum* and *P. nodosum*

**DOI:** 10.3390/plants12234033

**Published:** 2023-11-30

**Authors:** Silvana Moreno, Girma Bedada, Yousef Rahimi, Pär K. Ingvarsson, Anna Westerbergh, Per-Olof Lundquist

**Affiliations:** Linnean Centre for Plant Biology, Department of Plant Biology, BioCenter, Swedish University of Agricultural Sciences, SE-750 07 Uppsala, Sweden; silvana.moreno@slu.se (S.M.); girma.bedada@slu.se (G.B.); yousef.rahimi@slu.se (Y.R.); par.ingvarsson@slu.se (P.K.I.); anna.westerbergh@slu.se (A.W.)

**Keywords:** accessions, aerenchyma, anatomy, forage grass, perennial, root

## Abstract

Timothy (*Phleum pratense*) is a cool-season perennial forage grass widely grown for silage and hay production in northern regions. Climate change scenarios predict an increase in extreme weather events with fluctuating periods of high rainfall, requiring new varieties adapted to waterlogging (WL). Wild accessions could serve as germplasm for breeding, and we evaluated the responses of 11 wild and 8 domesticated accessions of timothy, *P. nodosum* and *P. alpinum* from different locations in northern Europe. Young plants at tillering stage were exposed to WL for 21 days in a greenhouse, and responses in growth allocation and root anatomy were studied. All accessions produced adventitious roots and changed allocation of growth between shoot and root as a response to WL, but the magnitude of these responses varied among species and among accessions. *P. pratense* responded less in these traits in response to WL than the other two species. The ability to form aerenchyma in the root cortex in response to WL was found for all species and also varied among species and among accessions, with the highest induction in *P. pratense*. Interestingly, some accessions were able to maintain and even increase root growth, producing more leaves and tillers, while others showed a reduction in the root system. Shoot dry weight (SDW) was not significantly affected by WL, but some accessions showed different and significant responses in the rate of production of leaves and tillers. Overall correlations between SDW and aerenchyma and between SDW and adventitious root formation were found. This study identified two wild timothy accessions and one wild *P. nodosum* accession based on shoot and root system growth, aerenchyma formation and having a root anatomy considered to be favorable for WL tolerance. These accessions are interesting genetic resources and candidates for development of climate-resilient timothy varieties.

## 1. Introduction

*Phleum pratense* L. is a perennial grass growing at higher latitudes in Northern Europe, East Asia and North America [1]. Its domesticated form is known as timothy and is widely cultivated for grazing and production of hay and silage. It is highly palatable for cattle and horses, having a high nutritive value and digestibility [2,3]. In Scandinavia, timothy is the dominant forage grass due to its winter hardiness, rapid growth and high biomass production. It is grown in pure stands or in mixtures with other perennial forage grasses such as *Festuca pratensis*, as well as perennial legumes such as red and white clover. Forage crops are cultivated on about 40% (>1 million hectares) of the agricultural land in Sweden, which is about the same size as the area used for the cultivation of annual cereal crops [4].

Timothy is an outcrossing species. High polymorphism and large genetic diversity have been found within cultivars [5]. Although timothy is perennial, it is short-lived. It has a shallow root system, and new tillers are formed from buds in the crown and swollen stem bases. These superficially lying meristems are easily damaged by cattle tramping and grazing, particularly in wet soils. Timothy is cultivated in both mesic and drier meadows but is considered to be sensitive to waterlogged soils, drought and heat [6,7,8].

The production of timothy is challenged by the ongoing climate change. Fluctuations in precipitation intensity with longer extreme wet and dry periods are expected to be more frequent [9,10,11], and increased rainfall and snowmelt in winter will cause stressful conditions for overwintering perennial plants [12]. Intense precipitation increases the risk of water saturation in the soil, leading to oxygen (O_2_) deficiency. The deficiency of O_2_ limits root and microorganism respiration and nutrient cycling, leading to reduced uptake of mineral nutrients by the plant roots [13,14,15], which negatively influences root and shoot growth [16]. Due to reduced N uptake and photosynthetic capacity and degradation of chlorophyll, responses by plants to waterlogging (WL) are also associated with leaf chlorosis and senescence [17,18]. Some plants have the ability to form extended gas-filled cavities in the shoots, called lacunae, and in the roots, called aerenchyma, under WL. These anatomical structures facilitate the diffusion of oxygen to organs that suffer from O_2_ deficiency, as well as transport CO_2_ and toxic volatiles from the roots [19,20,21,22].

Aerenchyma is formed specifically in the root cortex, but not in other root tissues such as the epidermis, hypodermis, endodermis or stele. It is formed through programmed cell death and the degradation of cortical cells, particularly in adventitious roots. The formation of adventitious roots increases the exchange of gases and absorption of nutrients and is also an adaptation to WL stress [23]. Certain plant species form aerenchyma as part of their normal development in drained soil [24], but its formation is particularly promoted when plants are exposed to flooding conditions [25,26,27]. A greater and faster capacity to form aerenchyma can enhance flooding tolerance [28,29]. Other anatomical root traits, such as a narrow stele and a large root diameter, may also contribute to improving WL tolerance [30,31]. The stele may be the first region at which hypoxia is sensed [22,32]. The stele of thin roots is the pathway of water movement into the central vascular tissues. A narrower stele and larger cortex have been found to be associated with an increased development of aerenchyma [31].

Tolerant plants can develop additional morphological changes, such as barriers to radial O_2_ loss (ROL), created by suberization and/or lignification of the cell walls in the outer cell layers of the roots [33,34,35,36,37]. Physiological adaptations, such as changes in photosynthesis and biochemical mechanisms involving endogenous hormone signals, play a central role in WL tolerance [38,39,40,41]. Knowledge about adaptations and mechanisms in WL tolerance in monocots is mostly known from studies of annual cereals and less from perennial forage grasses. For timothy, waterlogging tolerance has been addressed in relation to hardening and freezing tolerance [42,43], but there are no studies to date on the phenotypic diversity among accessions and possible tolerance mechanisms.

Although changes in crop and soil management may help to reduce yield losses caused by WL, the development of tolerant crops is urgently needed to achieve a highly stable crop production and sustainable agriculture. Wild relatives of crops make up a potential gene pool of traits and genes that are important for the development of climate-resilient cultivars. While crops are shaped by human selection to produce high yields in homogenous agricultural environments, their wild relatives are adapted to various habitats and geographical regions across the species range. Possible fluctuations in the intensity and direction of natural selection over time and across habitats and geographical sites have shaped the genetic structure of wild populations, and large genetic and phenotypic diversity may be found within wild crop relatives [44,45,46,47]. Moreover, intense directional selection during the domestication process has reduced the genetic diversity in domesticated crops, and traits and genes have been left behind in the wild relatives [48,49]. We hypothesize that there is variation in response to WL among wild and among domesticated accessions of *P. pratense* and related species, and that tolerant accessions can be found in wild populations and serve as genetic resources for improving WL tolerance in timothy.

*P. pratense* and its related species turf timothy, *P. nodosum* (syn. P. pratense L. subsp. bertolonii (DC.) Bornm. and P. bertolonii (DC.) Bornm.), and alpine timothy, *P. alpinum* L., commonly grow in the Nordic countries. Wild *P. pratense* is adapted to meadows and human-impacted and disturbed soils at low elevations throughout the Nordic countries. *P. alpinum* is commonly found at high elevations on meadows, riverbanks and roadsides, and in birch forests in Iceland, Norway, northern Sweden and northern Finland. *P. nodosum* has a more restricted and southern distribution than *P. alpinum* and is found in human-impacted habitats at low elevations. These species differ in ploidy level. *P. pratense* is hexaploid (2n = 6x = 42), *P. nodosum* is diploid (2n = 2x = 14) and *P. alpinum* L. is tetraploid (2n = 4x = 28). The phylogenetic relationships between these three species and other *Phleum* species are not yet fully understood. However, similarities have been found between two of the genomes of *P. pratense* and the diploid *P. nodosum* genome. Genetic similarities have also been found between *P. pratense* and *P. alpinum* [50,51]. *P. nodosum* and *P. alpinum* may therefore have had a direct or indirect role in the evolution of *P. pratense*, and it is therefore relevant to study the diversity of phenotypic responses among accessions of these related species.

We investigated the response to WL in domesticated and wild accessions from different habitats and latitudes. To identify tolerant accessions, we compared the performance of the same accessions in WL and non-waterlogging (NWL) cultivation by studying their shoot and root growth, their root anatomy and their ability to form aerenchyma.

## 2. Results

### 2.1. Plant Morphology and Growth among Phleum Species in NWL

An overall comparison of the three species in NWL showed that *P. pratense* and *P. nodosum* had higher growth than *P. alpinum. P. pratense* had a similar shoot dry weight (SDW) to *P. nodosum* but a significantly higher SDW than *P. alpinum* at the end of the experiment (*p* < 0.05, ANOVA, Table 1). The morphology/growth habit differed significantly among the species since, for both tiller number (TN) and leaf number (LN) per plant, *P. nodosum* showed the highest values, followed by *P. pratense* and *P. alpinum* (Figure S1). To further investigate the morphology and growth habit, several ratios between SDW, TN and LN as well as the exponential growth rate constants for the relative growth rates of TN and LN (RGR-TN, RGR-LN) were calculated.

TN:SDW and LN:SDW showed that the values for *P. nodosum* were 2-fold and 3-fold higher than those of *P. pratense*, respectively. *P. nodosum* formed a higher number of leaves per tiller (LN:TN) than the other species.

**Figure 1:**
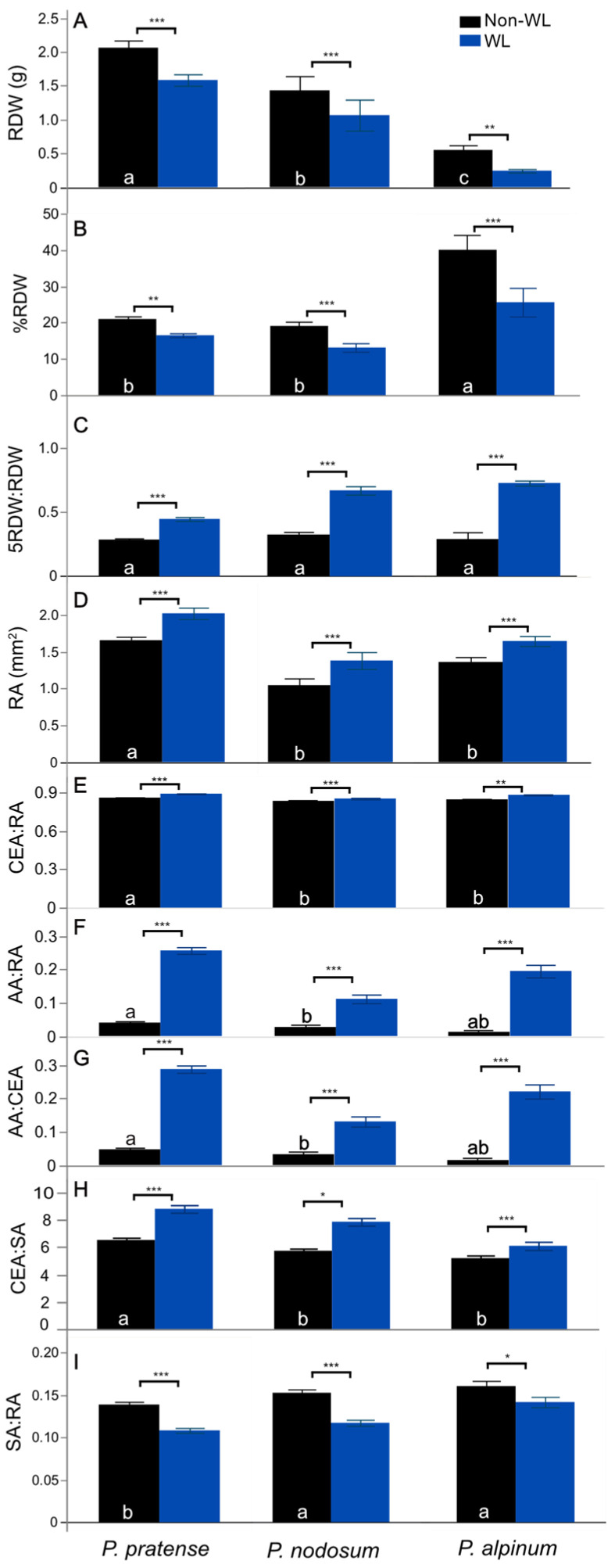
Root traits in *P. pratense*, *P. nodosum* and *P. alpinum* at 21 days in waterlogging treatment. (**A**) Root dry weight (RDW). (**B**) Percent root weight of total plant dry weight (%RDW). (**C**) Ratio of dry weight of the upper 5 cm of the root system and the dry weight of the total root (5RDW:RDW). The root anatomy traits: (**D**) root cross section area (RA). (**E**) Ratio of the cortex area to root cross section area (CEA:RA). (**F**) Ratio of aerenchyma to root cross section area (AA:RA). (**G**) Ratio of aerenchyma to cortex area (AA:CEA). (**H**) Ratio of the cortex to stele (CEA:SA). (**I**) Ratio of the stele area to root cross section area (SA:RA). Values are means and error bars are standard error (±SE). Significant differences between treatments within each species are represented by * = *p* < 0.05, ** = *p* < 0.01 and *** = *p* < 0.001 according to ANOVA. Differences among species in NWL are indicated by different letters when significantly different according to ANOVA and Tukey’s method, *p* < 0.05.

RGR-TN and RGR-LN were highest in *P. nodosum* and lowest in *P. alpinum* in NWL (*p* < 0.05, ANOVA, Table 1). *P. alpinum* had the lowest TN and LN at the start of the experiment, and it also showed the smallest increase in numbers of tillers and leaves in NWL during the experiment (ΔTN and ΔLN), as well as the increase expressed as percentage (%TN and %LN) (Figure S2). Plant growth allocation to root biomass was similar in *P. pratense* and *P. nodosum*, but higher in *P. alpinum* (Figure 1B). The species also differed in root anatomy, where *P. pratense* had a significantly higher root cross section area (RA) and ratio of area of cortex including epidermisto RA (CEA:RA), and lower ratio of stele to RA (SA:RA) than *P. nodosum* and *P. alpinum* (Figure 1D–I). Also, *P. pratense* had higher ratios of aerenchyma area to RA (AA:RA), AA:CEA and CEA:SA than *P. nodosum*, where most of the accessions did not form aerenchyma in NWL (Table S5).

### 2.2. Effects of WL on Growth Traits

#### 2.2.1. Comparisons at the Species Level

Comparisons at the species level showed that WL resulted in lower TN:SDW for *P. pratense* but not for the other two species (Table 1). None of the other shoot traits were significantly different in response to WL for any species. The percentage of growth allocation to roots (%RDW) decreased for all species in WL (Figure 1B), *P. alpinum* being the most affected (Table S6). Interestingly, the proportion of root biomass in the upper 5 cm part of the root system (5RDW:RDW) was ca. 30% in NWL for all species, while in WL, it was significantly higher at 45%, 65% and 71% for *P. pratense*, *P. nodosum* and *P. alpinum*, respectively (*p* < 0.05, ANOVA, Figure 1C). *P. pratense* responded significantly less to WL in the trait 5RDW:RDW compared to *P. nodosum* and *P. alpinum* (Table S6).

None of the accessions in any species showed chlorotic leaves in WL. Generative tillers were produced in both NWL and WL conditions (61% and 74% of all plants, respectively) and by all accessions except one for *P. pratense* (Table S3). All accessions of *P. nodosum* formed generative tillers in NWL and WL (75% and 80% of all plants, respectively), while none of the *P. alpinum* accessions had generative tillers.

#### 2.2.2. *P. pratense* Accessions

There was a large variation among accessions for SDW, ΔTN, ΔLN, RGR-TN, RGR-LN, TN and LN, as well as the ratios TN:SDW, LN:SDW and LN:TN, with 1.5- to 3-fold differences between the highest and lowest values in all traits (*p* < 0.05, ANOVA, Table 2, Tables S2 and S3) for both treatments. TN:SDW was the only shoot trait that showed a significant difference in response to WL (*p* < 0.05, ANOVA, Table 2).

The variation among accessions was not related to the type of group of accession (wild and domesticated) for the shoot traits (*p* > 0.05, ANOVA, Table 2). Between the two groups of accessions, there was a significant difference in response to WL in TN:SDW and LN:SDW (*p* < 0.05, ANOVA, Table 2). The accession W1 differed from the other *P. pratense* accessions by having a significantly higher number of leaves in WL, seen as LN:TN, as well as higher RGR-LN in WL (*p* < 0.05, Tables S2 and S3). Also, accession D1 produced significantly fewer leaves in WL.

**Figure 2:**
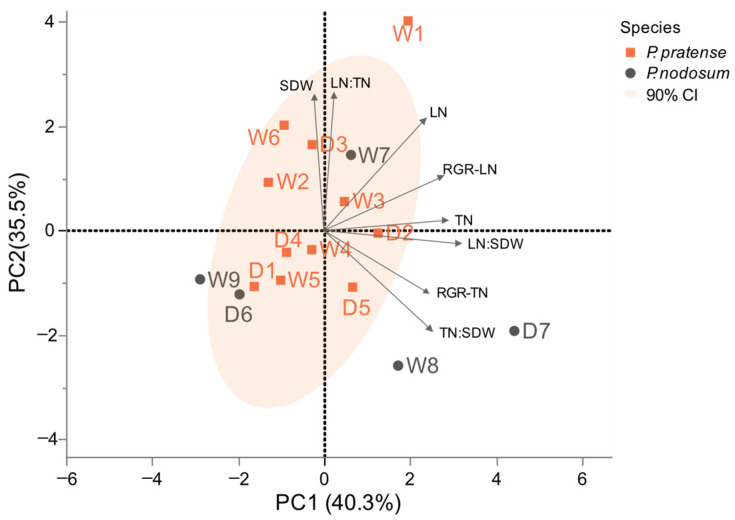
PCA based on the shoot dry weight, tiller and leaf production and growth rates of accessions of *P. pratense* and *P. nodosum*. *P. alpinum* was excluded due to no development of generative tillers. Data points represent the difference between WL and NWL. The loadings are shown for the variables of the shoot dry weight (SDW), tiller number (TN), leaf number (LN), the ratios of the tiller number to shoot dry weight (TN:SDW), the leaf number to shoot dry weight (LN:SDW), the leaf number per tiller (LN:TN) and the growth rates of the tiller number (RGR-TN) and leaf number (RGR-LN). The ellipse corresponds to the 90% confidence interval.

From a production perspective, the shoot biomass based on leaf and tiller numbers is crucial. Different growth and morphology of accessions and their responses to WL taken together could highlight possible traits relevant for sustainable yields. Therefore, to further analyze responses by all accessions of *P. pratense* and *P. nodosum*, we performed a principal component analysis (PCA) on the difference between the WL and NWL of all measured and calculated shoot traits (Figure 2). This showed that accessions grouped based on the shoot dry weight, tiller and leaf production. Accessions W1, W6 and D3 can be suggested to be more tolerant to waterlogging based on the correlation for the shoot traits stimulated by waterlogging. In contrast, the accessions W5 and D1 are more affected by WL.

**Figure 3:**
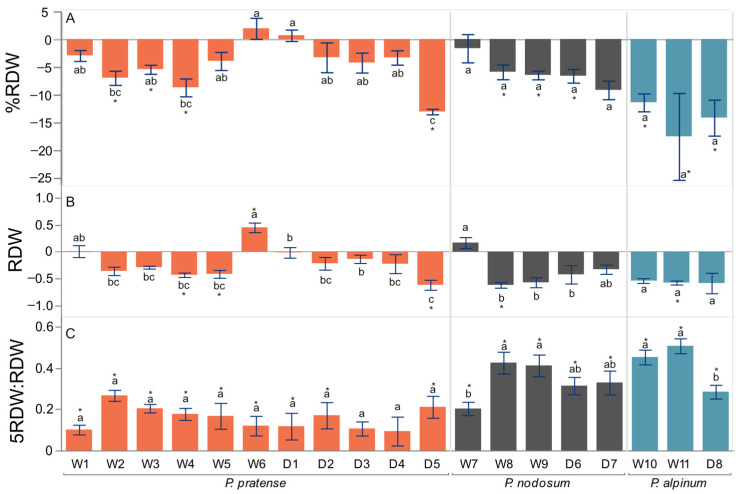
Effect of waterlogging on root traits of accessions. (**A**) Absolute difference between WL and NWL in percent root weight of total plant dry weight (%RDW). (**B**) Proportional difference in total root dry weight (RDW). (**C**) Absolute difference in ratio of the dry weight of the upper 5 cm of the root and the dry weight of the total root (5RDW:RDW) in wild (W) and domesticated (D) accessions of *P. pratense*, *P. alpinum* and *P. nodosum*. Values are means and error bars are ±SE. Significant differences between WL and NWL according to *t*-test are shown with *. Mean values that do not share the same letter are significantly different among accessions within each species according to ANOVA and Tukey’s method, *p* < 0.05.

For the root traits, there was a significant difference among accessions and between treatments in RDW, %RDW and 5RDW:RDW (*p* < 0.05, ANOVA, Table 2). There was also an interaction between accessions and treatments in RDW and %RDW. For traits that showed significant effects of WL based on ANOVA, we tried to resolve and display differences among accessions by calculating absolute or proportional differences (Figure 3A–C). For the absolute difference in %RDW, accessions W2, W3, W4 and D5 showed a significantly lower response to WL (*p* < 0.05, Figure 3A, Table S4). Root biomass was higher in the upper part of the root system in WL for W2, W3, W4, W5, W6, D1, D2 and D5, seen as a significant absolute difference in 5RDW:RDW (*p* < 0.05, Figure 3C, Table S4). This increased between 1.3- and 1.9-fold.

#### 2.2.3. *P. nodosum* Accessions

For *P. nodosum*, the response pattern for the accessions was similar to what was found for *P. pratense*. There was a significant difference among accession for all the traits, except for the TN:SDW (*p* < 0.05, ANOVA, Table 2 and Table S2), and no significant response to WL for all shoot traits (*p* > 0.05). There was a variation in shoot traits of 1.5- to 3-fold differences between the highest and lowest values (*p* < 0.05, ANOVA, Table 2 and Table S3) irrespective of the treatment. Accession D6 showed a significantly lower number of leaves (LN and ΔLN) in response to WL (*p* < 0.05, ANOVA, Tables S2 and S3).

The results of the PCA showed that accession W7 exhibited indications of tolerance to waterlogging due to the positive correlation with shoot traits that are enhanced by waterlogging (Figure 2). On the contrary, accessions W9 and D6 displayed greater susceptibility. Notably, the *P. nodosum* accessions W8 and D7 can be considered tolerant based on tiller traits stimulated by waterlogging.

For the root traits, there was a significant difference among accessions and between treatments in RDW, %RDW and 5RDW:RDW (*p* < 0.05, ANOVA, Table 2). The accessions W8, W9 and D6 allocated less biomass to the root system (%RDW) in WL (*p* < 0.05, Figure 3A, Table S4). All accessions of *P. nodosum* showed an increase in growth on the upper part of the root system in response to WL, since the absolute difference in 5RDW:RDW was significantly higher (*p* < 0.05, Figure 3C, Table S4).

#### 2.2.4. *P. alpinum* Accessions

For the *P. alpinum* accessions, SDW, LN:SDW and LN:TN, there were significant differences among accessions (*p* < 0.05, ANOVA, Table 2). In WL, the number of leaves per tiller increased for accession W11 (*p* < 0.05, ANOVA, Table S2) and the RGR-LN decreased for accession W10 (*p* < 0.05, ANOVA, Table S3).

In WL, all accessions allocated less biomass to the root system (%RDW) (*p* < 0.05, Figure 3A, Table S4) and showed increased growth in the upper part of the root system (5RDW:RDW) (*p* < 0.05, Figure 3C, Table S4).

### 2.3. Effects of WL on Root Anatomy

#### 2.3.1. Comparisons at the Species Level

**Figure 4:**
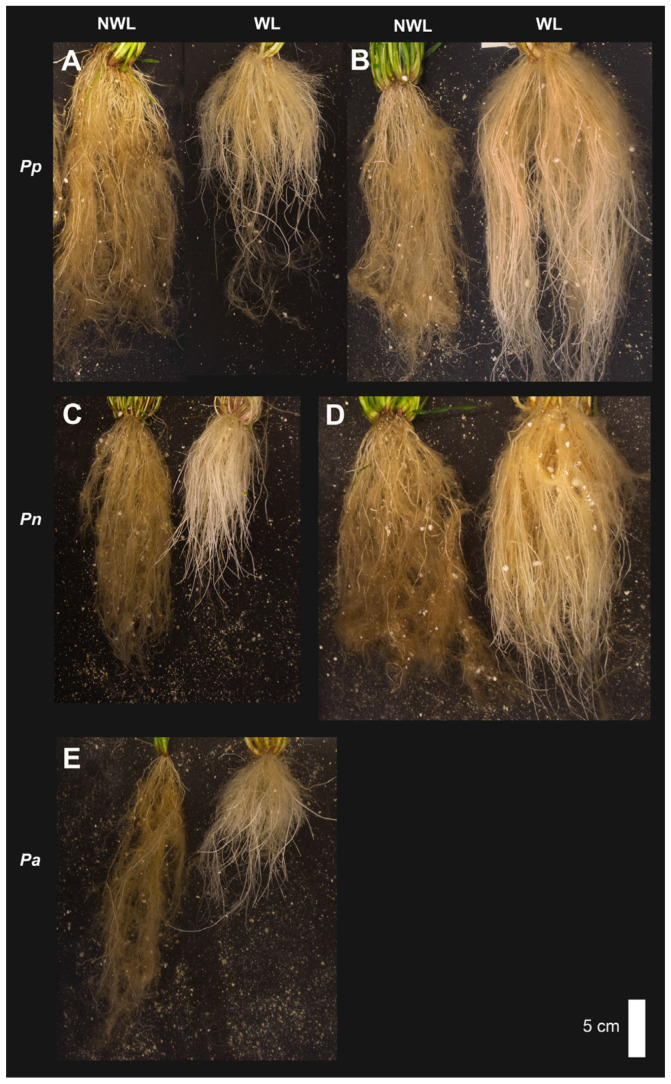
Root systems of representative plants showing different responses in non-waterlogging conditions (NWL, plants to the left side) and in waterlogging conditions (WL, plants to the right side). (**A**) *P. pratense*, accession D4, (**B**) *P. pratense*, accession W6, (**C**) *P. nodosum*, accession W9, (**D**) *P. nodosum*, accession W7 and (**E**) *P. alpinum*, accession W10. Bar is 5 cm.

The anatomy of the roots was also affected by the WL treatment (Figure 4). Significant differences between treatments were observed in RA, CEA:RA, AA:CEA, SA:RA and CEA:SA for all species (*p* < 0.05, ANOVA, Table 2). Interestingly, the formation of aerenchyma was several-fold higher in WL, as seen in the increase in the AA:RA and AA:CEA (Figure 1F,G). The CEA:SA was 1.2- to 1.4-fold higher in WL.

#### 2.3.2. *P. pratense* Accessions

**Figure 5:**
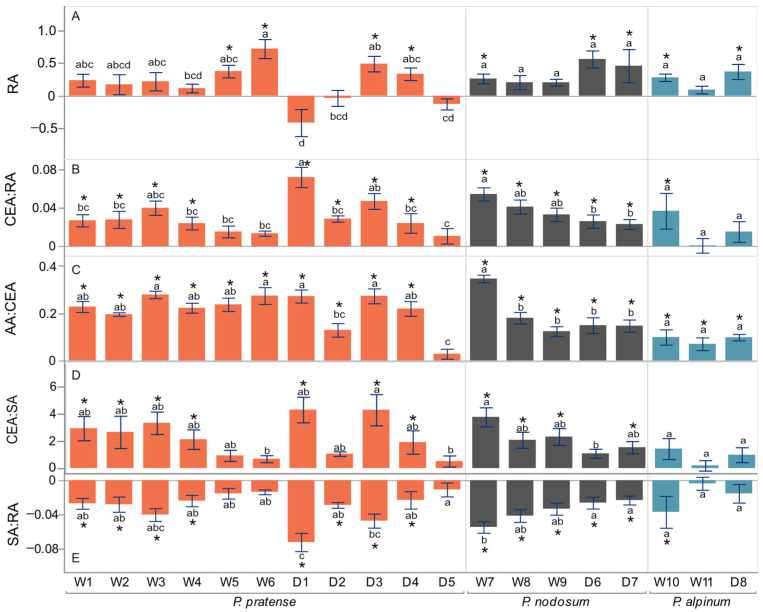
Effect of waterlogging on root anatomy in wild (W) and domesticated (D) accessions of *P. pratense*, *P. nodosum* and *P. alpinum*. (**A**) Proportional difference between WL and NWL in the root cross section area (RA). (**B**) Absolute difference in the ratio of the cortex area to root cross section area (CEA:RA) (**C**) Absolute difference in the ratio of the aerenchyma to cortex area (AA:CEA). (**D**) Absolute difference in the cortex to stele area (CEA:SA). (**E**) Absolute difference in the ratio of the stele to root cross section area (SA:RA). Values are means and error bars are ±SE. Significant differences between WL and NWL according to *t*-test are shown with *. Mean values that do not share the same letter are significantly different among accessions within each species according to ANOVA and Tukey’s method, *p* < 0.05.

RA and SA varied among accessions and in response to WL (Figure 5A,E, Table S5). Accessions W5, W6, D3 and D4 showed a significant increase in the RA in WL. Accession W6 had a significantly bigger SA, while accession D1 had a significantly smaller SA in WL. All accessions of *P. pratense* formed aerenchyma as part of their normal development in NWL. They showed a strong increase in response to WL since the AA:CEA was significantly higher in WL in all accessions except D5 (Figure 5C, Table S5). Accessions W1, W2, W3, W4, D1, D2, D3 and D4 had a slightly higher CEA:RA and lower SA:RA in WL.

There was a significant difference between the two groups of accessions (wild and domesticated) and a significant interaction between treatments and groups for AA:CEA (*p* > 0.05, ANOVA, Table 2). Between the two groups, there were significant differences in response to WL in RA, CEA:RA, AA:RA, AA:CEA and SA:RA (*t*-test *p* < 0.05, Figure S5).

#### 2.3.3. *P. nodosum* Accessions

Accession W7 of *P. nodosum* formed aerenchyma in NWL and WL conditions at a similar level as *P. pratense*, while the remaining accessions only formed aerenchyma in WL (Figure 5A–E, Table S5). Accession W7 had a 2-fold higher RA and SA than other *P. nodosum* accessions in WL and NWL. The absolute difference in response to WL in CEA:RA and SA:RA was significant for all accessions and followed a pattern similar to that of *P. pratense*.

#### 2.3.4. *P. alpinum* Accessions

Accessions of *P. alpinum* had between 3- and 6-fold more aerenchyma in the cortex than in NWL (Figure 5C, Table S5). Accessions W10 and D8 showed significantly thicker roots (Figure 5A, Table S5). In addition, W10 showed a significantly larger cortex (CEA:RA) and smaller stele (SA:RA) in WL (Figure 5B,E, Table S5).

### 2.4. Relations of Root Anatomy and Growth Traits

To try to understand which traits can be beneficial for tolerance to waterlogging and provide a basis to identify potential tolerant accessions, we analyzed the data for correlations among traits based on all data. Pearson correlation analysis showed that all shoot traits had strong positive correlations between each other in both treatments (Table 3). The 5RDW:RDW was negatively correlated to SDW (Table 3, Figure S6) and RDW in WL, suggesting that plants allocate resources to the production of more adventitious roots, which also has an effect on shoot development.

There was no correlation between the shoot and the root anatomical traits in NWL, while in WL the CEA:RA, AA:RA and AA:CEA were positively correlated to the shoot traits and RDW. In WL, the RA and the proportions of aerenchyma (AA:RA, AA:CEA) and cortex (CEA:RA) were negatively correlated to 5RDW:RDW, suggesting that plants having high formation of aerenchyma or a thick cortex, allocated less growth to the upper part of the root system.

The SA:RA was negatively correlated to the shoot traits in WL but not in NWL, suggesting that a smaller stele is beneficial for tolerance to waterlogging. The SA:RA was positively correlated to 5RDW:RDW, suggesting that plants that have a smaller stele area allocate their growth to the upper part of the root system. RA did not correlate to shoot traits but to RDW in both treatments.

To explore interactions of root anatomical traits and accession diversity, we performed a principal component analysis (PCA) on the areas and ratios of the root tissues (Figure S3). The results showed that the two components (PC1 and PC2) explained 94.4% of the variation. AA:CEA, CEA:SA, and SA:RA contributed strongly to PC1, while RA and SA contributed strongly to PC2. Most of the *P. pratense* accessions in WL correlated to AA:CEA and CEA:SA and separated from the NWL (Figure S3A). In WL, the domesticated accessions showed a large diversity in response, while the wild accessions showed a more similar response in root anatomy (Figure S3B). In NWL, the accessions of domesticated and wild *P. pratense* formed two partially overlapping clusters. In NWL, four out of five *P. nodosum* accessions were different from *P. pratense* and *P. alpinum*, and distributed with a negative correlation to SA and RA. The fifth *P. nodosum* accession (W7) clustered together with the *P. pratense* accessions in both NWL and WL.

**Figure 6:**
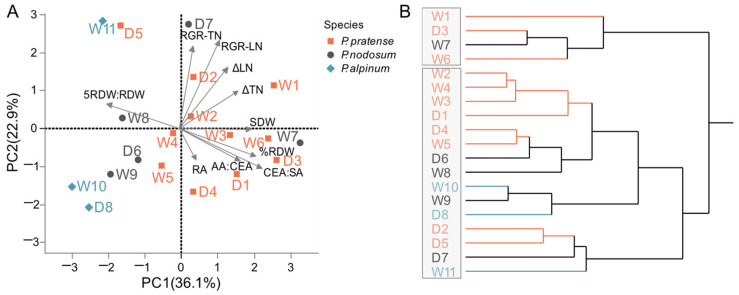
Distribution of accessions of *P. pratense*, *P. nodosum* and *P. alpinum* based on differences between WL and NWL in the shoot and root traits and the root anatomy traits. (**A**) PCA where data points represent an accession. For measured traits (SDW, ΔTN, ΔLN and RA), proportional differences between WL and NWL were used, and for the remaining calculated traits (RGR-TN, RGR-LN, %RDW, 5RDW:RDW, AA:CAE, CAE:SA), the absolute differences were used. (**B**) HCA based on the same traits as for the PCA. Abbreviations can be found in Table 2.

To display variation in tolerance among accessions, we performed a PCA and an HCA on the proportional or absolute difference in response to WL for shoot and root traits, growth rates of leaf and tiller, and root anatomy traits (Figure 6). SDW, % RDW, AA:CEA and CEA:SA contributed positively, and 5RDW:RDW contributed strongly and negatively to PC1 (36.1%). TN, LN and the growth rates of LN (RGR-LN) and TN (RGR-TN) contributed to PC2 (22.9%) in the PCA (Figure 6A). The response to WL of the accessions W1, W6, W7 and D3 correlated to the SDW, % RDW, AA:CEA and CEA:SA and this distribution was supported by the HCA (Figure 6B). Accession D5 (Tryggve) was strongly affected by 5RDW:RDW and negatively correlated to the other anatomy traits.

To analyze possible differences in the timing of response to WL, we performed a PCA on weekly growth rates of tiller and leaf numbers of all accessions (Figure S4). Accessions showing a positive correlation with the loading variable had a higher growth rate in WL. Some accessions were grouped depending on the first week response to WL (R-LN7, R-TN7), while others grouped along the axis influenced by the second (R-LN14, R-TN14) or third (R-LN21, R-TN21) week variables.

R-LN7 and R-TN7 contributed strongly and positively to PC1. A high positive value along the first week variables showed that growth in WL was higher than in NWL. In contrast, those accessions placed in the opposite direction of these variables were negatively affected by the WL condition in the first week. The direction of R-LN21 and R-TN21 variables corresponded to a higher rate of formation of tillers and leaves in WL. Accessions W1, W4, W5, W8, W11 and D4 were more affected during the first week; however, their growth increased in the last week of WL conditions.

## 3. Discussion

Timothy is one of the most cultivated forage crops in northern Europe, and new cultivars adapted to a changing climate are needed to secure harvest yields. While drought is predicted to be the main abiotic stress for plants in central and southern Europe, more frequent and heavy rainfalls, with flooded and waterlogged soils as a result, are predicted to be the main challenge in northern Europe. The effect of WL stress on annual cereals such as barley and wheat has been well studied, but few studies have focused on perennial forage grasses in general and timothy and other *Phleum* species in particular.

Our aim was to evaluate timothy and two related species for their responses to WL and to investigate tolerance mechanisms, which have not been previously described in *Phleum* species. By evaluating a large set of accessions, we discovered variation in growth allocation, root growth and root anatomy, suggesting possible mechanisms of tolerance to waterlogging and how they vary among accessions and species. These results provide valuable knowledge for the selection of WL-tolerant accessions as possible inclusions in breeding based on their phenotypic responses and as candidates for further genetic studies.

To compare species and accessions, it is valuable to know their morphologies and growth rates under a normal irrigation regime. The results show that the three species differ clearly in growth habit and morphology, e.g., number of tillers and leaves per plant (Table S3, Figure S1). Interestingly, one *P. nodosum* accession appeared to be closer to *P. pratense* in these shoot traits (Figure S1), as well as in root anatomy traits (Figure S3). The taxonomy of *Phleum* is challenging, with many species and subspecies [51], and the phenotypic relatedness of this *P. nodosum* accession to *P. pratense* points out the importance of studying the diversity of *Phleum* gene bank collections.

Both *P. pratense* and *P. nodosum* developed reproductive tillers in both WL and NWL. In future studies on the effects of WL under field conditions, forage quality and seed production should be investigated, as it has been shown that WL has a negative effect on these traits in annual cereals [52,53,54] and forage crops [55,56,57]. *P. alpinum* differed from the other species since all accessions remained in the vegetative stage under these growth conditions (Table S3). This may be related to the high temperatures in the greenhouse and partly to the daylength, since *P. alpinum* has been shown to require a dual induction by low temperature and/or short day to induce flowering, as well as a combination of long days and high temperatures to enhance heading and inflorescence development [58,59].

The response to WL with regards to SDW, compared at the species level with data from all accessions combined, showed no significant differences between waterlogged and non-waterlogged plants in the three species (Table 1). Previous studies in perennial grasses, using a similar length of WL treatment as in this study, have reported tolerant varieties of cocksfoot, tall fescue and perennial ryegrass that showed no significant difference in SDW in WL and NWL [60,61]. In annual cereals, such as wheat and barley, exposure to WL affected the leaf appearance, shoot biomass and flowering [62,63,64]. Even short periods of WL in young wheat plants have significant long-term effects and cause reduced plant growth and development [65]. It is clear from our experiment that the negative effects of WL on shoot biomass seen in annual monocots were not observed in any of the three *Phleum* species. This suggests that *P. pratense*, *P. nodosum* and *P. alpinum* are relatively tolerant to WL compared to annuals and have evolved mechanisms to cope with this stress. To compare the three species, WL response indexes for several traits were calculated (Table S6), and the indexes for %RDW and for formation of root biomass in the upper part of the root system (5RDW:RDW) suggest that *P. pratense* responded less to WL than the other two species.

A major objective of this study was to investigate the diversity among *Phleum* accessions in how they respond to WL. Interestingly, we found that among the several accessions in this study, there was a variation in WL tolerance (Table 2), as well as between type of accession and between species. In some accessions, growth was negatively affected by WL (*P. pratense* D1, *P. nodosum* D6, *P. alpinum* W10); interestingly, in contrast, some other accessions responded by producing more leaves per tiller (*P. pratense* W1, *P. alpinum* W11), and for W1, there was a higher growth rate in leaf formation (RGR-LN). The timing of the response in the growth rate of tillers and leaves also appeared to vary among accessions, with some being affected in the first week and others in the second or third week of WL (Figure S4).

Root development and growth were clearly affected by WL (Figure 4), and in general, the three species showed a decrease in root biomass. This is a common response in plants, as shown for annual cereals [52,66,67,68], annual dicot crops [69,70], perennial forage grasses [61,71] and perennial forage legumes [72,73]. Root growth decreases due to a deficiency of oxygen, energy, nutrients and accelerated root senescence [74].

Interestingly, among the accessions, there seem to be different types of responses in distribution of the root and shoot biomass (Figure 4). Some accessions, perhaps less tolerant, showed a decrease in the proportion of root biomass (%RDW) in response to WL (*P. pratense* W3 and W4, *P. nodosum* W8 and W9, *P. alpinum* W10). In contrast, accessions *P. pratense* W6 and *P. nodosum* W7 had an increase in RDW or no effect on RDW, as for *P. pratense* W1. These accessions showed no significant effect on %RDW (Figure 3A), consistent with the fact that shoot growth was not negatively affected (Figure 2). The response by W1, W6 and W7 could be part of a mechanism to escape WL by the root as well as the shoot. It may resemble the response to escape WL through shoot elongation, which is a common flood and submergence response of many wetland plants and crops, where it serves to restore contact with the atmosphere and sustain internal aeration [75,76].

The increase in RDW in WL for the two accessions W6 and W7 could be the result of continued root growth, less senescence and higher formation of adventitious roots. In all accessions of the three species, the root abundance in the 5 cm section below the root:shoot interface (5RDW:RDW) increased in WL due to the formation of adventitious roots (Figure 3C). The formation of new adventitious roots with aerenchyma can increase oxygen availability in the roots and nutrient uptake from the soil, enabling roots to grow [21,77]. Anatomical adaptations such as aerenchyma formation, thick roots, large cortex to stele areas and the growth of adventitious roots improve WL tolerance [78,79]. All *P. pratense* and *P. alpinum* accessions and one accession of *P. nodosum* formed aerenchyma in a low percent of the root cortex in NWL conditions. Formation of aerenchyma in NWL conditions has been also reported in other perennial grasses, e.g., roughstalk bluegrass, tall fescue and cocksfoot [60,80]. However, it can be increased in hypoxic conditions, as we observed in most accessions. Some accessions showed the ability to form more aerenchyma than others (Figure 5C). We found a high positive correlation between the shoot dry weight (SDW) and the ratio between aerenchyma and cortex (AA:CEA) in WL (Table 3), suggesting the importance of aerenchyma formation for WL tolerance in *Phleum* also.

Another important objective was to test the hypothesis that there are differences between accessions in WL tolerance and, if possible, suggest new germplasm for breeding. A tolerant plant that is desirable from a breeding perspective should have a high capacity to form adventitious roots and aerenchyma, a high cortex to stele ratio and the ability to maintain shoot and root growth. Combining shoot and root traits (Figure 6), accessions W1, W6, W7 and D3 may be considered more tolerant than the other accessions due to a higher production of aerenchyma in the cortex (AA:CEA), higher CEA:SA and maintained shoot and root dry weight. A maintained and even increased root growth, as seen in accessions W6 and W7, might be advantageous for a rapid recovery after WL, as was also suggested for WL-tolerant species [81]. In contrast, accession D5 had a smaller root system with a higher proportion of roots in the upper 5 cm region (5RDW:RDW) and produced aerenchyma at the same low level as in NWL, suggesting that D5 (cv. Tryggve, common cultivar in Sweden) may be less tolerant to WL under the experimental conditions. The response of producing higher 5RDW:RDW could be in line with an increased production of the number of adventitious roots in WL as an adaptation to WL, which was shown for cocksfoot (*Dactylis glomerata*) [54]. For further pre-breeding studies, we suggest including the wild accessions of *P. pratense* W1 and W6 and *P. nodosum* W7.

WL is an important ecological factor, and species adapted to flooded habitats commonly form aerenchyma in response to WL [22]. Root anatomies with high CA:SA and AA:CA were also suggested to be advantageous for WL tolerance [41]. The accession passport information, available for most of the wild accessions in the present study (Table S1), shows that, e.g., some of the less tolerant accessions, such as W3 and W9, were collected from areas with comparatively less precipitation (www.smhi.se) and habitats described as meadow and grassland. In contrast, two of the more tolerant accessions, W1 and W6, were collected from habitats described as bog and marsh, and these accessions have a root anatomy favorable for WL tolerance. Differential strategies within a species to tolerate flooding depending on the habitat from where the populations originate has been found for other plant species (e.g., [82,83]), supporting the importance of WL as a selective ecological factor.

Comparing wild and domesticated accessions of *P. pratense* made it possible to test the hypothesis that there are differences between these two groups of accessions. In NWL, there is a difference in the cortex (CEA:RA, CEA:SA) and stele (SA:RA) areas between the domesticated and wild groups of accessions. Interestingly, among the wild accessions, the accessions collected in wetlands, W1 and W6, had the smallest root cross-sectional and stele areas, as well as the highest CEA:SA. High cortex to stele area is one of the root anatomical factors that explain the adaptation of wild grasses to high soil water content [84].

Since a two-way ANOVA showed significant differences in root anatomical traits (Table 2) between the two groups, the root anatomical trait data were further tested for absolute or proportional differences between WL and NWL plants (Figure S5). For the response to WL in RA, CEA:RA, AA:RA, AA:CEA and SA:RA, the wild and domesticated accessions were significantly different (*t*-test, *p* > 0.05). The significant differences for some of these traits were largely due to accession D5. When D5 was excluded from the *t*-test analyses, the significant difference between the wild and domesticated groups remained in the response in the CEA:RA and SA:RA traits. Our results showed that SA:RA was significantly lower in WL for both wild and domesticated accessions, but the reduction during WL was lower for the wilds (Figure S5). Also, in NWL, the group of wild accessions had lower SA:RA compared to the domesticated accessions. The xylem makes up a large proportion of the stele (SA), and it has been found that for plants adapted to wet soils, the xylem to the whole root area is generally lower [84].

WL results in low O_2_ availability for the root system and thus affects respiration, metabolism and transport of mineral nutrients and water. Tolerance to WL is therefore a complex trait needed for sustained growth and depends on several plant adaptations in physiology, metabolism and gene expression. Our study has demonstrated a diversity in phenotypic responses of growth and root anatomy to WL in young plants of *P. pratense*, *P. nodosum* and *P. alpinum* at the tillering stage. A reduction in root system and a production of adventitious roots was observed in most of the accessions, while a few accessions also maintained root growth. Based on plant growth traits, aerenchyma formation and other root anatomical traits, we could identify wild accessions of *P. pratense* and *P. nodosum* that could be tested further for use in breeding programs. In screening for WL-tolerant timothy, the studied root morphology and anatomy traits could be valuable when identifying tolerant offspring. Further studies are required to elucidate the molecular mechanisms underpinning WL tolerance in *Phleum* species.

## 4. Materials and Methods

### 4.1. Plant Material

The performance of 244 wild and domesticated accessions of *P. pratense*, *P. nodosum* and *P. alpinum* was evaluated in the field and greenhouse in Uppsala, Sweden [85]. A total of 19 accessions that represented the variation within species were selected for the present study of the response to WL. Six wild accessions and five cultivars of *Phleum pratense* (timothy), three wild accessions and two cultivars of *P. nodosum*, and two wild accessions and one breeding line of *P. alpinum* from the Nordic countries were used (Table S1). Seeds were obtained from the gene bank NordGen, Alnarp, Sweden, except for the cultivars Tryggve and Switch, provided by Lantmännen, Svalöv, Sweden.

### 4.2. Pre-Cultivation

Seeds of each accession were sown in 2 L pots with a mixture of vermiculite and perlite (1:1) in a greenhouse at the Plant Cultivation Facility, Uppsala BioCenter, SLU, Uppsala, Sweden, with a 16/8 hrs day/night photoperiod with supplementary light from metal halogen lamps. The experiment was carried out from February to April 2022, at a maximum temperature of 24 °C and a minimum of 16 °C. For each accession, three seeds per pot were sown in eight pots. Plants were given a complete nutrient solution (N:102 mg/L (NH_4_^+^: 40 mg/L, NO_3_^−^: 62 mg/L), P: 20 mg/L, K: 86 mg/L, S: 8 mg/L, Ca: 6 mg/L, Mg: 8 mg/L, Fe: 0.34 mg/L, Mn: 0.4 mg/L, B: 0.2 mg/L, Zn: 0.06 mg/L, Cu: 0.03 mg/L, Mo: 0.08 mg/L; Wallco Miljöcenter AB, Arlöv, Sweden) every third day. Three weeks after sowing, eight plants of each accession of similar size were selected by removing the other plants in each pot.

### 4.3. WL Experiment

After seven weeks in pre-cultivation, four of the eight plants of each accession were exposed to WL. All plants were at vegetative stage at the start of the experiment. Each pot was placed in a 5 L bucket. Deionized water and 200 mL of the complete nutrient solution were added up to the surface of the vermiculite and perlite mixture. The water level was maintained 1 cm above the soil line by gently adding deionized water. The other four plants were grown in NWL and were watered every third day with 300 mL of the complete nutrient solution. The plants were divided into four blocks, where one plant in WL and one plant in NWL of each accession were randomized in each block. The experiment was conducted for 21 days.

### 4.4. Studied and Calculated Traits

**Figure 7:**
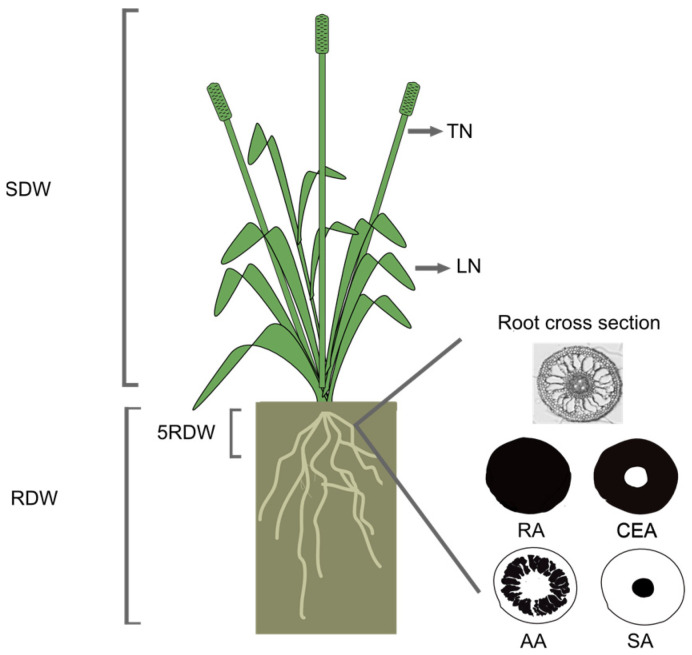
Drawing of the studied shoot and root traits and their abbreviations; shoot dry weight (SDW), number of tillers (TN) and number of leaves (LN) per plant, root dry weight (RDW), dry weight of the upper 5 cm part of the root system (5RDW), area of the root cross section (RA), stele area (SA), cortex area including epidermis (CEA) and aerenchyma area (AA). Measured tissue areas in the root anatomy are indicated in black.

The tiller number (TN) and leaf number (LN) of each plant (Figure 7) were counted at the start of the experiment and at 7, 14 and 21 days of WL. The number of tillers and leaves produced during the experiment (ΔTN and ΔLN) were calculated by subtracting the number of tillers and leaves at day 0. The TN and LN values for the time points 0, 7, 14 and 21 were used to calculate the growth rates by linear regression based on their natural logarithmic values (ln), where the slopes ln(TN) day^−1^ and ln(LN) day^−1^ represent the relative growth rates RGR-TN and RGR-LN, respectively. For weekly growth rates, the difference between time points was used.

At the end of the experiment, each plant was taken out from the pot and the roots were gently rinsed with tap water to remove the vermiculate and perlite substrate. We then divided the shoot and the root by cutting the plant at the interface between the two tissues. The shoot was dried at 65 °C for 48 h and the shoot dry weight (SDW) was then measured for each plant.

At harvest, two adventitious roots emerging from the crown were randomly selected from each plant and stored in 70% ethanol at 4 °C for anatomical analysis. Cross sections of the maturation zone of the roots (10–30 mm from the root base) were cut by hand. These root sections were visualized at 10× magnification using a bright-field Zeiss Axio Scope A1 microscope, and images were taken by an AxioCam ICc5 camera. The area of the root cross section (RA), the stele area (SA), the cortex area including epidermis (CEA) and the aerenchyma area (AA) were measured in ImageJ v. 1.52t (National Institute of Health, Bethesda, MD, USA) using the free hand tool.

After sampling for root anatomy studies, the root systems were cut 5 cm below the interface between the shoots and the roots. The two root parts were kept separately and dried at 65 °C for 48 h. Then, the dry weight of the upper 5 cm of the root (5RDW) and the total dry weight of the root were measured. The percentage of allocation of plant growth to roots, %RDW, was calculated as 100 × RDW:(RDW + SDW).

### 4.5. Analysis of Phenotypic Responses to WL

This study comprised wild and domesticated accessions of three different *Phleum* species. The non-treated accessions showed large diversity in shoot and root traits both within and between the species (Tables S2 and S3). To be able to compare the responses of different accessions, we calculated the absolute difference between WL and NWL conditions as

$${\bar{x}}_{WL}-{\bar{x}}_{NWL}=\frac{1}{n}\sum_{i=1}^{n}\left({x_{i}-\bar{x}}_{NWL}\right)$$

and the proportional difference as

$$\frac{{\bar{x}}_{WL}-{\bar{x}}_{NWL}}{{\bar{x}}_{NWL}}=\frac{1}{n}\sum_{i=1}^{n}\left({x_{i}-\bar{x}}_{NWL}\right)/\;{\bar{x}}_{NWL}$$

where ${\bar{x}}_{WL}$ is the average in WL, ${\bar{x}}_{NWL}$ is the average in the NWL and $x_{i}$ is the value for the individual *i* in WL.

The absolute difference was calculated for %RDW and the ratios LN:SDW, TN:SDW, LN:TN, RGR-TN, RGR-LN, 5RDW:RDW, CEA:RA, AA:RA, AA:CEA, CEA:SA and SA:RA. For the SDW, RDW, ΔTN, ΔLN and RA, the proportional difference was used.

### 4.6. Statistical Analysis

All traits showed normally distributed residuals and were therefore analyzed with the parametric test Analysis of Variance (ANOVA) using a Mixed Effects Model approach. The mixed model analysis was performed using restricted maximum likelihood estimation (REML) and unbounded variance components. For each *Phleum* species, the fixed effects included accessions and treatments (WL and NWL) and the interaction between them, while block was considered as random effects. For comparison of the wild and domesticated groups of *P. pratense* accessions, the fixed effects included group, treatment and the interaction between treatment and group. For comparisons between WL and NWL by accession, a subdesign ANOVA was performed considering the same fixed and random effects as the initial model. To compare the response to WL among accessions, a Tukey test was performed. Moreover, Pearson correlation analysis was applied to evaluate the linear association between traits. Data of growth traits used for Pearson correlation analysis were natural logarithm transformed. The multivariate analysis principal component analysis (PCA) and hierarchical cluster analysis (HCA) using Ward’s method were used to analyze and display the variation in response among accessions based on all studied shoot and root traits. Statistical significance was determined at *p* < 0.05. All statistical analyses were performed using JMP Pro 16.0.

## 5. Conclusions

WL of soil leads to reduced agricultural plant productivity and is expected to increase due to more frequent extreme weather conditions caused by climate change. This study has demonstrated substantial diversity in the response to WL of a range of wild and domesticated accessions of *P. pratense*, *P. nodosum* and *P. alpinum*. The identification of specific traits, including the ability to maintain root growth, the development of adventitious roots and aerenchyma formation, suggests the potential for these features to serve as valuable screening criteria for identifying tolerant accessions. Moreover, the study has pinpointed two wild *P. pratense* and one wild *P. nodosum* accessions with traits that could be useful as genetic resources for improving cultivars in the future. WL tolerance has not historically been a conscious target of breeding programs for timothy, which instead focused on traits such as early heading. Our findings offer a pathway for future breeding efforts to enhance the resilience of timothy to waterlogged conditions, further underscoring the importance of continued research in this area.

## Figures and Tables

**Table 1:** Shoot traits in *P. pratense*, *P. nodosum* and *P. alpinum* in non-waterlogging (NWL) and waterlogging (WL) treatment. Values are means of plants within each species and standard error (±SE). Differences among species in NWL are indicated by different letters (a, b or c) when significantly different according to ANOVA and Tukey’s method, *p* < 0.05. No significant differences were found between treatments within each species (*p* < 0.05, *t*-test). Abbreviations of traits: shoot dry weight (SDW), tiller number (TN), leaf number (LN), ratio of the tiller number to shoot dry weight (TN:SDW), ratio of the leaf number to shoot dry weight (LN:SDW), leaf number per tiller (LN:TN), growth rates of the tiller number (RGR-TN) and leaf number (RGR-LN).

Species	*P. pratense*		*P. nodosum*		*P. alpinum*	
Traits	NWL	WL	NWL	WL	NWL	WL
SDW (g)	7.88 ± 0.38 ^a^	8.07 ± 0.37	6.23 ± 0.62 ^a^	6.02 ± 0.86	0.85 ± 0.17 ^b^	0.82 ± 0.12
TN (#plant^−1^)	23.34 ± 0.99 ^a^	21.05 ± 0.89	30.79 ± 3.05 ^b^	29.21 ± 2.32	5.09 ± 0.79 ^c^	5.18 ± 0.42
LN (#plant^−1^)	138.89 ± 7.23 ^a^	129.14 ± 6.01	227.16 ± 21.12 ^b^	185.37 ± 13.5	24.0 ± 2.95 ^c^	23.27 ± 2.72
TN:SDW (#plant × g^−1^)	3.24 ± 0.21 ^b^	2.76 ± 0.13	5.40 ± 0.44 ^a^	6.31 ± 0.79	6.89 ± 0.69 ^ab^	10.02 ± 3.11
LN:SDW (#plant × g^−1^)	18.26 ± 0.84 ^b^	16.57 ± 0.65	41.47 ± 4.59 ^a^	39.47 ± 4.03	33.93 ± 4.48 ^ab^	46.92 ± 17.38
LN:TN	6.06 ± 0.25 ^b^	6.20 ± 0.19	7.57 ± 0.39 ^a^	6.7 ± 0.40	4.9 ± 0.40 ^b^	4.42 ± 0.30
RGR-TN (#day ^−1^)	0.052 ± 0.002 ^b^	0.050 ± 0.002	0.061 ± 0.002 ^a^	0.062 ± 0.004	0.036 ± 0.006 ^c^	0.027 ± 0.004
RGR-LN (#day ^−1^)	0.072 ± 0.002 ^b^	0.072 ± 0.002	0.092 ± 0.003 ^a^	0.087 ± 0.003	0.047 ± 0.004 ^c^	0.041 ± 0.004

**Table 2:** Two-way ANOVA results showing the effect of accession, treatment and their interaction in the different *Phleum* species, as well as the effect of type of accession (wild (W) or domesticated (D)), treatment and their interaction in *P. pratense* in non-waterlogging (NWL) and waterlogging (WL) conditions. * = *p* < 0.05, ** = *p* < 0.01, *** = *p* < 0.001. Abbreviations: shoot dry weight (SDW), tiller number (TN), leaf number (LN), ratio of the tiller number to shoot dry weight (TN:SDW), ratio of the leaf number to shoot dry weight (LN:SDW), leaf number per tiller (LN:TN), tiller number produced during the treatment (ΔTN), leaf number produced during the treatment (ΔLN), growth rates of the tiller number (RGR-TN) and the leaf number (RGR-LN), root dry weight (RDW), percent root weight of total plant dry weight (%RDW), ratio of dry weight of the upper 5 cm of the root system and the dry weight of the total root (5RDW:RDW), root cross section area (RA), ratio of cortex area to root cross section area (CEA:RA), ratio of aerenchyma to cortex area (AA:CEA), ratio of cortex to stele area (CEA:SA), ratio of stele area to root cross section area (SA:RA).

Source	SDW	TN	LN	TN:SDW	LN:SDW	LN:TN	ΔTN	ΔLN	RGR-TN	RGR-LN	RDW	%RDW	5RDW:RDW	RA	CEA:RA	AA:CEA	CE:SA	SA:RA
*P. pratense*																		
*Accessions*	2.5 **	2.5 **	3.7 ***	3.6 ***	5.3 ***	2.9 ***	2.3 *	3.7 ***	2.2 *	2.4 **	4.4 **	2.6 **	2.2*	4.2 ***	9.2 ***	3.8 ***	6.5 ***	9.1 ***
*Treatment*	0.1	3.4	1.6	5.3 *	3.8	0.2	4	1.6	1.1	0	21.2 ***	32.0 ***	85.9 ***	17.2 ***	78.6 ***	565.2 ***	66.0 ***	76.8 ***
*Accessions x Treatment*	0.5	0.5	1.2	1	1.3	1.5	0.4	1.3	0.9	1.5	3.6 ***	2.5 **	0.9	2.6 ***	2.4 **	5.7 ***	2.2 **	2.4 **
*P. nodosum*																		
*Accessions*	13.0 ***	6.9 ***	6.8 ***	2.1	4.3 ***	4.0 ***	5.6 **	6.4 ***	5.9 **	11.5 *	19.4 ***	6.0 ***	6.22 ***	50.3 ***	4.8 **	23.2 ***	2.3	4.1 **
*Treatment*	0	0	2.7	0.2	1	2.3	0	3.3	0.2	1.1	4.9 *	18.9 ***	126.1 ***	22.3 ***	55.5 ***	229.2 ***	5.7 *	53.9 ***
*Accessions x Treatment*	1.8	1	1.5	0.5	0.6	0.3	1.2	1.6	2	1.8	2.3	0.8	1.9	0.8	1.4	9.4 ***	1	2.4
*P. alpinum*																		
*Accessions*	6.5 **	0.1	3.2	2.6	4.7 ***	22.2 ***	0.2	2.7	0.5	0.3	0.4	13.0 ***	0.6	1	3.5 **	0.3	4.1 **	3.4 *
*Treatment*	0.1	0	0.1	1.1	0.6	2.7	0	0.3	0.1	1.1	14.2 **	12.7 ***	50.4 ***	10.9 ***	4.4 **	29.2 ***	53.9 ***	4.9 *
*Accessions x Treatment*	0.1	2.1	1.8	1.6	1.1	1.7	1.4	2.6	0.4	2.6	0.1	0.2	1.2	1.2	1.5	0.4	2.4	0.3
*W and D of P. pratense*																		
*Group*	1.5	2.7	1.1	3.9	2.6	0.1	0.8	0.6	1	0	1.7	0	1.1	14.5 ***	36.6 ***	394.4 ***	54.1 ***	54.1 ***
*Treatment*	0.1	0.9	0.5	5.5*	6.7 **	0.1	3.5	1.1	0.8	0.7	12.0 ***	22.1 ***	75.1 ***	0.4	65.3 ***	0.6	19.8 ***	19.8 ***
*Group x Treatment*	0.1	0.3	0.2	1.8	0.1	3.2	0.1	0.2	0	0.5	0.2	0	0.6	1.8	3.1	5.5 *	0.5	0.5

**Table 3:** Pearson correlation coefficient (r) for shoot and root traits for all accessions in non-waterlogging (NWL) and waterlogging (WL, in grey) conditions. Abbreviations: shoot dry weight (lnSDW), tiller number (lnTN), leaf number (lnLN), root dry weight (lnRDW), percent root weight of total plant dry weight (%RDW), ratio of dry weight of the upper 5 cm of the root system and the dry weight of the total root (5RDW:RDW), root cross section area (RA), ratio of cortex area to root cross section area (CEA:RA), ratio of aerenchyma to root cross section area (AA:RA), ratio of aerenchyma to cortex area (AA:CEA), ratio of cortex area to stele area (CEA:SA) and ratio of stele area to root cross section area (SA:RA).

	WL	ln(SDW)	ln(TN)	ln(LN)	ln(RDW)	%RDW	5RDW:RDW	RA	CEA:RA	AA:RA	AA:CEA	CEA:SA	SA:RA
NWL													
**ln(SDW)**			0.83	0.83	0.91	−0.47	−0.75	0.41	0.61	0.72	0.72	0.57	−0.61
**ln(TN)**		0.91		0.98	0.68	−0.49	−0.42	0.08	0.55	0.48	0.48	0.48	−0.55
**ln(LN)**		0.84	0.97		0.67	−0.48	−0.4	0.08	0.59	0.50	0.50	0.52	−0.59
**ln(RDW)**		0.87	0.72	0.65		−0.08	−0.88	0.59	0.58	0.83	0.83	0.56	−0.58
**%RDW**		−0.78	−0.78	−0.73	−0.4		−0.03	0.22	−0.24	0.01	0.01	−0.18	0.24
**5RDW:RDW**		−0.15	−0.02	−0.07	−0.41	−0.28		−0.69	−0.53	−0.79	−0.80	−0.53	0.53
**RA**		0.36	0.03	−0.06	0.64	0.08	−0.5		0.37	0.72	0.72	0.39	−0.37
**CEA:RA**		0.22	0.25	0.18	0.20	−0.12	0.08	0.11		0.74	0.72	0.98	−1.00
**AA:RA**		0.31	−0.03	−0.09	0.54	0.09	−0.47	0.90	0.08		1.00	0.73	−0.74
**AA:CEA**		0.30	−0.03	−0.09	0.54	0.09	−0.47	0.90	0.05	1.00		0.71	−0.72
**CEA:SA**		0.25	0.29	0.19	0.24	−0.14	0.05	0.12	0.97	0.05	0.02		−0.97
**SA:RA**		−0.24	−0.27	−0.19	−0.21	0.15	−0.09	−0.12	−1.00	−0.08	−0.06	−0.98	

## Data Availability

The data presented in this study are available on request from the corresponding author.

## Supplementary Materials

The following supporting information can be downloaded at https://www.mdpi.com/article/10.3390/plants12234033/s1. Figure S1. Relations among accessions of *P. pratense*, *P. nodosum* and *P. alpinum* based on morphological traits in non-waterlogging conditions (NWL) after 70 days of growth in greenhouse conditions. Figure S2. Growth of plants of *P. pratense*, *P. nodosum* and *P. alpinum* accessions shown as production of leaves and tillers during the waterlogging (WL) and non-waterlogging (NWL) treatments. Figure S3. Distribution and response of accessions of *P. pratense*, *P. nodosum* and *P. alpinum* to NWL and WL conditions analyzed using principal component analysis based on root anatomy traits. Figure S4. Diversity in effects of WL on weekly growth rates of tiller and leaf numbers. Figure S5. Effect of WL on root anatomy traits of domesticated and wild accessions of *P. pratense*. Figure S6. Correlations between shoot dry weight (SDW) and aerenchyma area to cortex–epidermis area (AA:CEA) and between SDW and proportion of dry biomass of upper 5 cm part of the root system (5RDW:RDW) of *Phleum* accessions in NWL and WL conditions. Table S1. Studied accessions and their passport data. Table S2. Shoot traits of *P. pratense*, *P. nodosum* and *P. alpinum* in NWL and WL at the end of the 21-day treatment. Table S3. Growth rates and generative tillers. Table S4. Root traits of *P. pratense*, *P. nodosum* and *P. alpinum* in non-waterlogging (NWL) and waterlogging (WL) at the end of the 21-day treatment. Table S5. Root anatomy traits of accessions of *P. pratense*, *P. nodosum* and *P. alpinum* in non-waterlogging (NWL) and waterlogging (WL) conditions at the end of the 21-day treatment. Table S6. WL response indexes of *P. pratense*, *P. nodosum* and *P. alpinum*, calculated as the proportional difference between the WL and NWL.
