# Supplementary material for: Response to Waterlogging Stress in Wild and Domesticated Accessions of Timothy (Phleum pratense) and Its Relatives P. alpinum and P. nodosum
Source: Plants (Basel). 2023 Nov 30;12(23):4033. doi: 10.3390/plants12234033 (PMC10708118; doi:10.3390/plants12234033)
Supplement: Supplementary file 1 [file plants-12-04033-s001.zip › plants-2670585-supplementary.pdf]

Supplementary materials to:

Response to waterlogging stress in wild and domesticated accessions of timothy (*Phleum pratense*) and its relatives *P. alpinum* and *P. nodosum*

Silvana Moreno, Girma Bedada, Yousef Rahimi, Pär K. Ingvarsson, Anna Westerbergh and Per-Olof Lundquist*

Linnean Centre for Plant Biology, Department of Plant Biology, BioCenter, Swedish University of Agricultural Sciences, Uppsala, Sweden

* Correspondence: Per-Olof.Lundquist@slu.se

Plants
https://www.mdpi.com/journal/plants

Section: Plant Response to Abiotic Stress and Climate Change
https://www.mdpi.com/journal/plants/sections/plant_response_abiotic_stress

Special Issue: Wild and Cultivated Plants under Climate Change
https://www.mdpi.com/journal/plants/special_issues/48YPV8QN2X

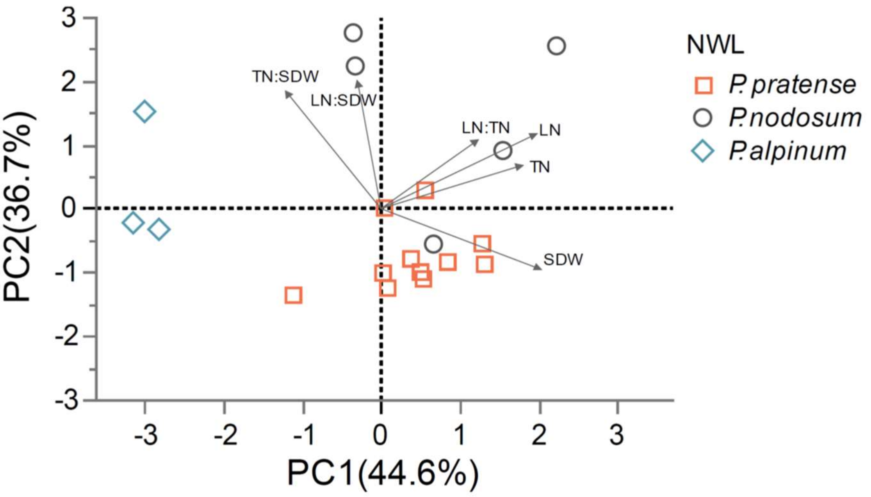

Figure S1. Relations among accessions of *Phleum pratense*, *P. nodosum* and *P. alpinum* based on morphological traits in non-waterlogging conditions (NWL) after 70 days of growth in greenhouse conditions. The data used for the principal component analysis is shoot dry weight (SDW), tiller number (TN) and leaf number (LN) per plant and their ratios.

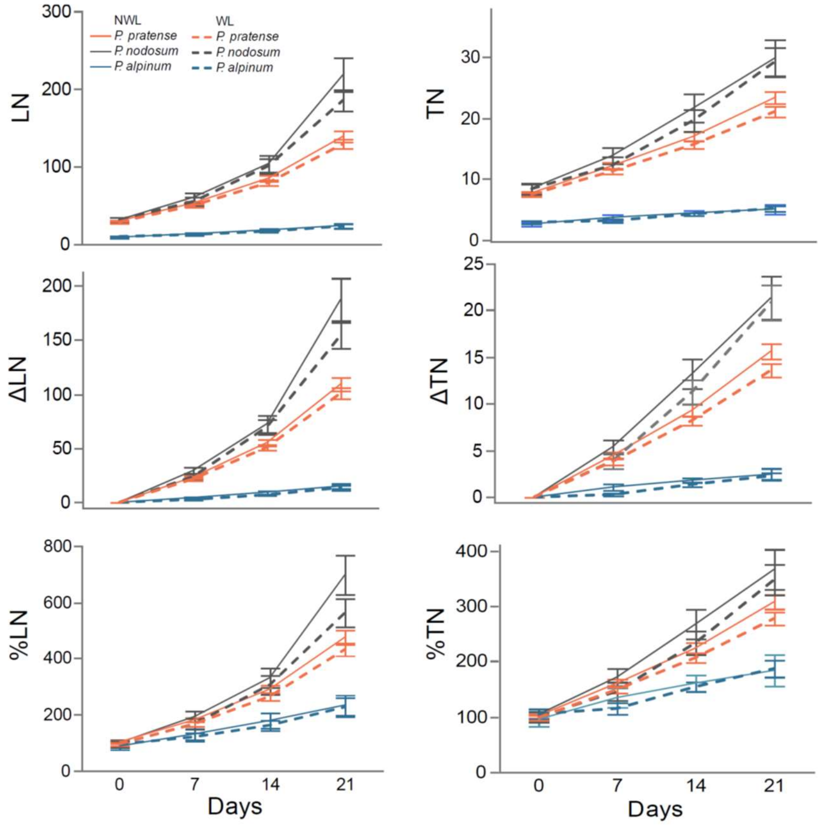

Figure S2. Growth of plants of *Phleum pratense*, *P. nodosum* and *P. alpinum* accessions shown as production of leaves and tillers during the waterlogging (WL) and non-waterlogging (NWL) treatments. The increase in number of leaves and tillers is shown as absolute number or as percentage increase in relation to number at the start of the experiment. The treatment started 49 days after germination. Data shown is averages for 11, 5 and 3 accessions for the three species respectively, with 4 replicate plants per accession. Error bars are standard errors.

A

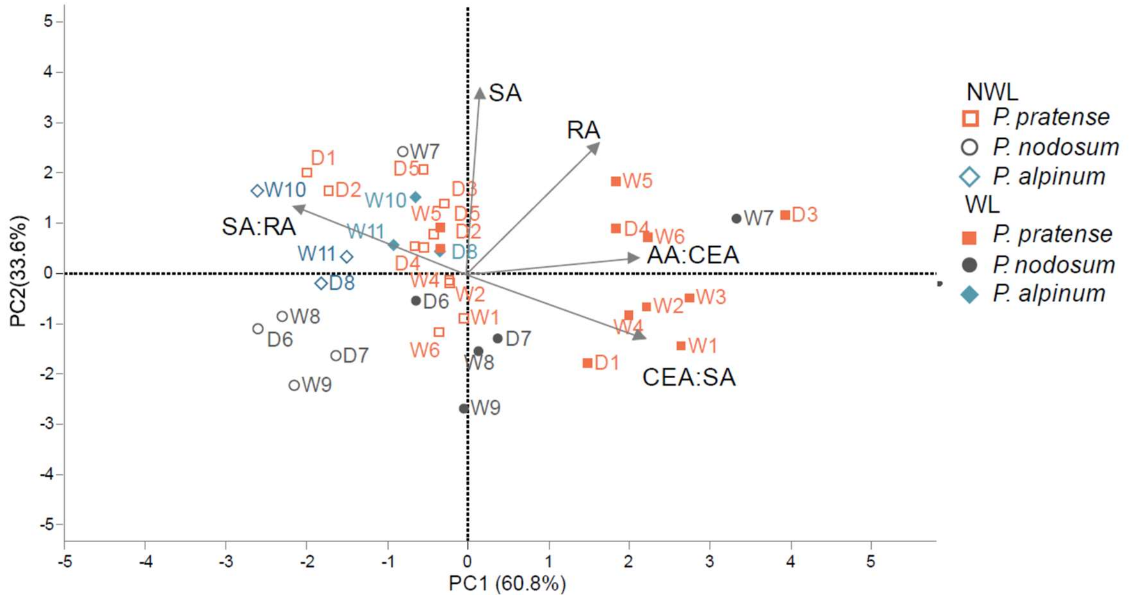

B

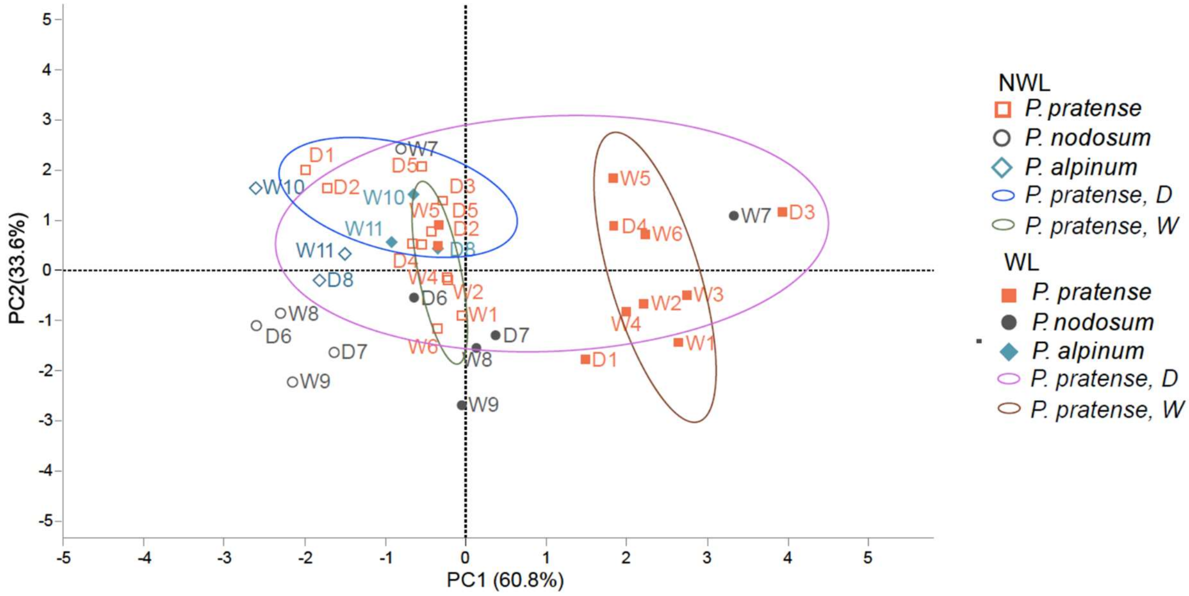

Figure S3. Distribution and response of accessions of *Phleum pratense*, *P. nodosum* and *P. alpinum* to non-waterlogging (NWL) and waterlogging (WL) conditions analyzed by principal component analysis based on root anatomy traits. Figure A shows the loadings of each anatomy variable (RA, root area; SA, stele area; and the ratios SA:RA; CEA:SA, cortex-epidermis area to stele area; and AA:CEA, aerenchyma area to cortex-epidermis area, together with the scores representing the accessions. Figure B shows the wild and domesticated accessions of *P. pratense* in WL and NWL where each group is circled by 90% confidence intervals.

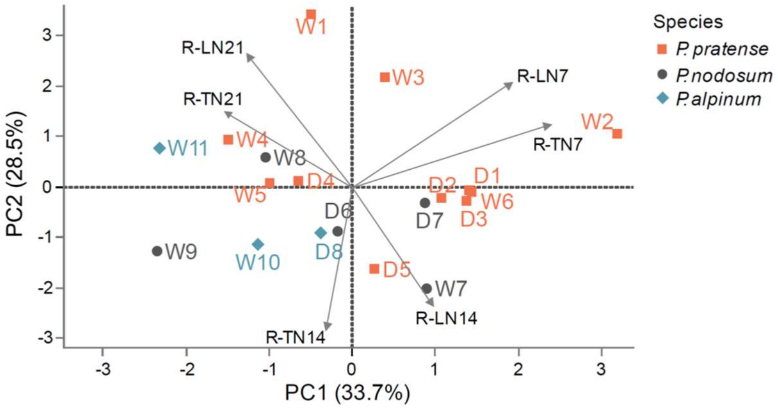

Figure S4. Diversity in effects of WL on weekly growth rates of tiller and leaf numbers. The calculated variables used for the principal component analysis are the differences in growth rate of tiller number (TN) and leaf number per plant (LN) for each accession between plants in WL and NWL during each of the first 7 days (7), the days 8-14 (14) and the days 15-21 (21) of the experiment. Accessions projected to be positively correlated to the loading of a variable had a higher growth rate in WL during that time period, and conversely, accessions negatively correlated to a variable loading had higher growth rate in NWL during that time period.

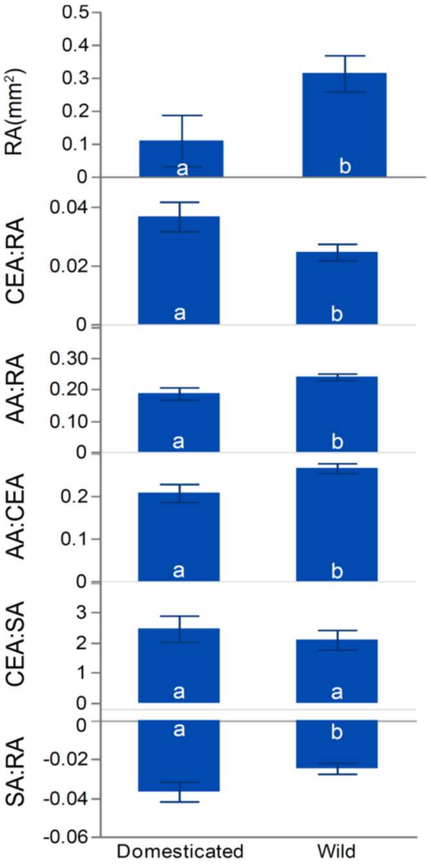

Figure S5. Effect of WL on root anatomy traits of domesticated and wild accessions of *Phleum pratense*. The effect was calculated as a proportional difference between plants in WL and NWL for root area (RA) and for absolute differences for the ratio variables. Domesticated and wild accessions indicated with different letters are significantly different ($p < 0.05$). Data shown is averages for 5 domesticated and 6 wild accessions, with 4 replicate plants per accession. Error bars are standard errors.

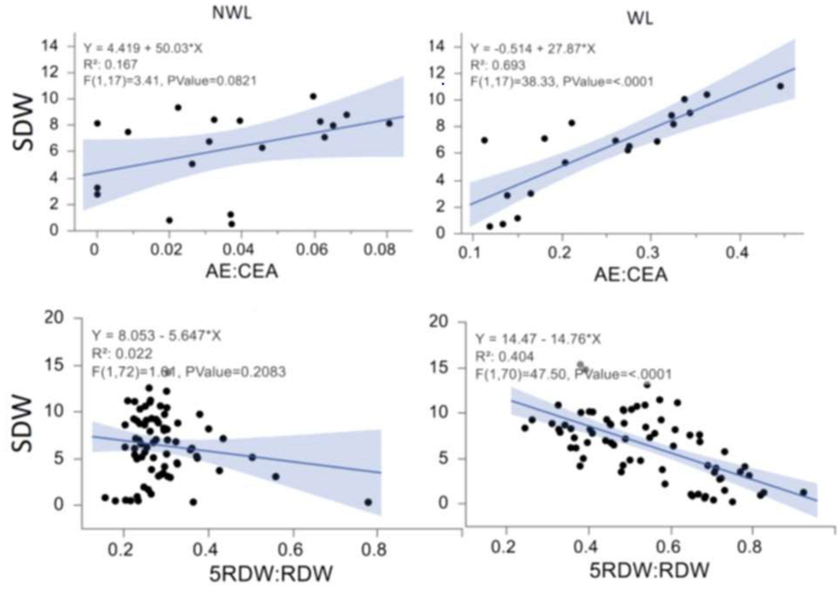

Figure S6. Correlations between shoot dry weight (SDW) and aerenchyma area to cortex-epidermis area (AA:CEA) and between SDW and the proportion of dry biomass of upper 5 cm part of the root system (5RDW:RDW) of *P. pratense*, *P. nodosum* and *P. alpinum* accessions in NWL and in WL conditions at the end of the 21 days treatment.

Table S1. Studied *Phleum* sp. accessions and their passport data.

Accession ID-SLU	Species	Accession number or cultivar	Type of accession	Habitat	Country of origin	Coordinates	Latitude	Longitude	Collection date
**W1**		NGB4053	Wild	Bog	Denmark	57.2417, 9.7750	57° 14' 30.12"	9° 46' 30"	08/26/1983
**W2**		NGB21082	Wild	Field margin	Norway	59.1158, 10.1971	59° 6' 56.88"	10° 11' 49.56"	8/21/2009
**W3**		NGB1537	Wild	Meadow	Sweden	57.4917, 18.1750	57° 29' 30.12"	18° 10' 30"	8/9/1985
**W4**		NGB722	Wild	Grassland	Sweden	66.6917, 19.7583	66° 41' 30.1194"	19° 45' 29.8794"	8/25/1979
**W5**		NGB20801	Wild	Field	Sweden	64.6644, -21.2133	64° 39' 51.84"	-21° 12' 47.88"	5/28/2008
**W6**	*Phleum pratense*	NGB7707	Wild	Marsh	Norway	67.9432, 15.2253	67° 56' 35.52"	15° 13' 31.08"	1/1/1976
**D1**		NGB13524	Domesticated		Sweden				
**D2**		NGB11677	Domesticated		Denmark				
**D3**		NGB13221	Domesticated		Sweden				
**D4**		Cultivar Switch	Domesticated		Sweden				
**D5**		Cultivar Tryggve	Domesticated		Sweden				
**W7**		NGB14477	Wild	Meadow/Road/Field/Ditch	Finland	60.1539, 23.0878	60° 9' 14.04"	23° 5' 16.08"	8/16/1998
**W8**		NGB4364	Wild	Grassland	Sweden	56.3250, 12.5250	56° 19' 30"	12° 31' 30"	9/2/1983
**W9**	*Phleum nodosum**	NGB4352	Wild	Grassland	Sweden	55.3917, 14.1583	55° 23' 30.12"	14° 9' 29.88"	8/17/1983
**D6**		NGB2378	Domesticated		Sweden				
**D7**		NGB1725	Domesticated		Denmark				
**W10**		NGB1340	Wild	Grassland	Sweden	64.4250, 18.0583	64° 25' 29.9994"	18° 3' 29.8794"	9/15/1980
**W11**	*Phleum alpinum*	NGB772	Wild	Grassland	Sweden	68.2083, 22.7750	68° 12' 29.8794"	22° 46' 29.9994"	8/24/1979
**D8**		NGB4143	Domesticated		Iceland				

**P. nodosum* (syn. *P. pratense* L. subsp. *bertolonii* (DC.) Bornm. and *P. bertolonii* (DC.) Bornm.).

Table S2. Shoot traits of *P. pratense*, *P. nodosum* and *P. alpinum* in non-waterlogging (NWL) and waterlogging (WL) at the end of the 21 days treatment. Shoot dry weight (SDW), number of tillers (TN), number of leaves (LN), the ratios of the number of tillers to shoot dry weight (TN:SDW), the number of leaves to shoot dry weight (LN:SDW), the number of leaves to the number of tillers (LN:TN). Values are means and standard error (± SE) of plants within each accession.

Accessions	SDW (g)		TN (#plant^{-1})		LN (#plant^{-1})		TN:SDW (#plant x g^{-1})		LN:SDW (#plant x g^{-1})		LN:TN	
	NWL	WL	NWL	WL	NWL	WL	NWL	WL	NWL	WL	NWL	WL
P. pratense												
**W1**	5.07 ± 0.76	6.25 ± 0.69	17.75 ± 2.93	18.00 ± 2.04	68.00 ± 9.68	110.50 ± 13.36	3.62 ± 0.56	2.88 ± 0.02	13.80 ± 1.67	17.89 ± 1.55	**3.90 ± 0.21**	**6.23 ± 0.57**
**W2**	6.30 ± 1.44	6.96 ± 0.85	24.50 ± 2.33	18.50 ± 2.50	147.00 ± 16.04	120.25 ± 19.54	**4.53 ± 1.12**	**2.71 ± 0.30**	**25.26 ± 2.91**	**17.51 ± 2.14**	6.14 ± 0.79	6.45 ± 0.25
**W3**	8.34 ± 1.46	9.04 ± 0.47	21.25 ± 2.29	22.50 ± 1.89	136.50 ± 26.73	145.25 ± 19.61	2.72 ± 0.38	2.51 ± 0.26	16.79 ± 2.04	16.22 ± 2.39	6.28 ± 0.56	6.39 ± 0.35
**W4**	6.76 ± 0.21	6.54 ± 0.53	31.75 ± 4.87	29.25 ± 3.12	182.25 ± 16.91	161.25 ± 19.60	4.66 ± 0.64	4.44 ± 0.20	26.86 ± 2.06	24.51 ± 1.89	5.92 ± 0.42	5.51 ± 0.28
**W5**	10.19 ± 0.78	8.20 ± 1.38	25.25 ± 1.03	19.50 ± 3.48	172.50 ± 20.07	124.50 ± 20.11	2.51 ± 0.17	2.56 ± 0.48	17.02 ± 1.84	15.47 ± 1.60	6.91 ± 1.01	6.73 ± 1.27
**W6**	8.42 ± 2.12	10.06 ± 1.45	25.00 ± 2.04	20.00 ± 1.87	134.75 ± 11.98	141.50 ± 16.15	**3.69 ± 1.12**	**2.06 ± 0.19**	18.01 ± 2.66	14.40 ± 1.09	5.55 ± 0.75	7.05 ± 0.41
**D1**	9.34 ± 0.53	8.86 ± 0.73	25.50 ± 2.66	20.25 ± 1.65	**182.75 ± 32.79**	**122.75 ± 14.35**	2.72 ± 0.21	2.29 ± 0.05	19.35 ± 2.65	13.80 ± 0.75	7.34 ± 1.38	6.02 ± 0.25
**D2**	8.28 ± 1.12	8.29 ± 0.88	18.50 ± 3.01	20.50 ± 1.44	126.50 ± 15.77	134.00 ± 10.27	2.26 ± 0.31	2.54 ± 0.26	15.39 ± 0.59	16.59 ± 1.67	7.10 ± 0.73	6.55 ± 0.28
**D3**	8.79 ± 0.94	10.41 ± 1.54	24.75 ± 4.37	24.00 ± 5.76	155.00 ± 21.00	166.25 ± 31.60	2.75 ± 0.24	2.23 ± 0.31	17.53 ± 1.27	15.71 ± 1.23	6.45 ± 0.46	7.28 ± 0.61
**D4**	7.09 ± 0.91	6.91 ± 1.70	19.75 ± 3.35	19.67 ± 3.18	113.50 ± 10.18	103.33 ± 15.07	2.91 ± 0.58	3.07 ± 0.47	16.80 ± 2.53	16.18 ± 2.50	6.01 ± 0.52	5.32 ± 0.50
**D5**	8.13 ± 1.64	6.99 ± 1.81	22.75 ± 2.81	19.00 ± 1.78	109.00 ± 22.01	84.50 ± 8.76	3.24 ± 0.91	3.14 ± 0.57	14.10 ± 2.50	13.86 ± 2.42	5.06 ± 1.06	4.44 ± 0.14
P. nodosum												
**W7**	7.98 ± 1.01	11.04 ± 1.58	25.00 ± 4.34	30.50 ± 5.24	159.25 ± 26.89	180.50 ± 15.99	3.15 ± 0.36	2.85 ± 0.51	19.78 ± 1.56	17.37 ± 3.06	6.41 ± 0.44	6.27 ± 0.81
**W8**	8.15 ± 1.23	5.31 ± 1.17	41.00 ± 7.43	35.50 ± 1.76	250.50 ± 40.51	196.75 ± 10.96	**5.06 ± 0.61**	**8.04 ± 2.08**	30.54 ± 0.57	41.85 ± 7.39	6.29 ± 0.70	5.62 ± 0.55
**W9**	3.26 ± 0.19	2.87 ± 0.58	21.50 ± 2.60	15.75 ± 2.14	189.00 ± 35.42	124.25 ± 16.53	6.79 ± 1.07	6.10 ± 1.06	59.91 ± 13.48	48.42 ± 9.87	8.76 ± 1.02	8.10 ± 1.02
**D6**	7.49 ± 0.99	7.12 ± 1.43	40.75 ± 7.70	35.50 ± 3.95	**341.00 ± 44.20**	**244.75 ± 35.17**	5.69 ± 1.05	5.63 ± 1.44	47.81 ± 7.37	35.63 ± 2.15	8.68 ± 0.85	7.08 ± 0.98
**D7**	3.61 ± 0.41	3.01 ± 0.57	24.00 ± 3.05	28.67 ± 5.49	185.66 ± 19.46	179.00 ± 40.58	6.64 ± 0.31	9.81 ± 1.81	51.91 ± 4.80	58.97 ± 3.28	7.79 ± 0.43	6.33 ± 0.88
P. alpinum												
**W10**	1.24 ± 0.36	1.16 ± 0.14	6.25 ± 1.93	4.50 ± 0.65	25.50 ± 6.76	19.00 ± 3.03	6.12 ± 1.79	4.14 ± 0.85	24.20 ± 5.29	16.91 ± 2.73	4.21 ± 0.35	4.29 ± 0.44
**W11**	0.52 ± 0.02	0.55 ± 0.16	4.00 ± 0.00	6.25 ± 0.48	25.75 ± 0.95	32.75 ± 1.93	7.79 ± 0.22	17.30 ± 7.42	50.28 ± 3.14	91.90 ± 40.86	**6.44 ± 0.24**	**5.29 ± 0.30**
**D8**	0.80 ± 0.25	0.72 ± 0.25	5.00 ± 1.53	4.67 ± 0.88	19.67 ± 7.31	16.33 ± 3.67	6.73 ± 1.16	8.15 ± 2.45	25.12 ± 3.55	26.97 ± 6.17	3.78 ± 0.26	3.44 ± 0.29

Data in bold indicates significant difference between NWL and WL ($p < 0.05$, sub-design ANOVA).

Table S3. Growth rates and generative tillers. Growth rates were calculated as produced number of tillers (ΔTN) and leaves (ΔLN) during the treatment, and exponential growth rates of the number of tillers (RGR-TN) and leaves (RGR-LN) in *P. pratense*, *P. nodosum* and *P. alpinum* in non-waterlogging (NWL) and waterlogging (WL) treatment. Values are means and standard error (± SE) of plants within each accession. Number of plants with generative tillers of the total number of plants per accession.

**Accessions**	**ΔTN (# plant^{-1})**		**ΔLN (# plant^{-1})**		**RGR-TN (# day^{-1})**		**RGR-LN (# day^{-1})**		**Number of plants with generative tillers**	
	**NWL**	**WL**	**NWL**	**WL**	**NWL**	**WL**	**NWL**	**WL**	**NWL**	**WL**
P. pratense										
**W1**	12.25 ± 2.29	11.25 ± 1.65	47.50 ± 7.90	89.00 ± 11.19	0.057 ± 0.003	0.046 ± 0.006	**0.057 ± 0.003**	**0.076 ± 0.005**	3/4	4/4
**W2**	16.75 ± 2.50	12.75 ± 2.06	119.25 ± 13.75	98.00 ± 17.13	0.056 ± 0.005	0.057 ± 0.009	0.077 ± 0.001	0.081 ± 0.005	2/4	4/4
**W3**	12.75 ± 1.03	14.50 ± 2.25	106.75 ± 20.56	113.50 ± 21.70	0.046 ± 0.004	0.047 ± 0.006	0.073 ± 0.004	0.070 ± 0.010	4/4	4/4
**W4**	22.75 ± 4.23	20.75 ± 2.95	144.50 ± 15.55	124.75 ± 15.25	0.061 ± 0.005	0.062 ± 0.007	0.076 ± 0.004	0.072 ± 0.005	0/4	0/4
**W5**	17.00 ± 1.41	12.25 ± 2.32	137.50 ± 14.01	98.00 ± 16.70	0.053 ± 0.006	0.048 ± 0.005	0.077 ± 0.006	0.072 ± 0.003	1/4	2/4
**W6**	15.75 ± 1.80	11.75 ± 2.10	104.25 ± 9.99	108.50 ± 12.98	0.046 ± 0.003	0.044 ± 0.007	0.071 ± 0.002	0.068 ± 0.003	2/4	2/4
**D1**	16.50 ± 2.33	12.00 ± 1.08	**147.75 ± 30.08**	**94.50 ± 10.20**	0.048 ± 0.004	0.043 ± 0.002	0.076 ± 0.004	0.069 ± 0.002	4/4	2/4
**D2**	12.00 ± 2.38	13.50 ± 1.32	98.75 ± 15.17	105.00 ± 6.75	0.046 ± 0.003	0.049 ± 0.004	0.070 ± 0.005	0.074 ± 0.003	3/4	3/4
**D3**	16.75 ± 3.33	14.75 ± 4.75	123.50 ± 16.18	132.50 ± 27.76	0.051 ± 0.003	0.041 ± 0.008	0.076 ± 0.004	0.075 ± 0.007	1/4	4/4
**D4**	14.75 ± 3.09	12.67 ± 1.33	93.50 ± 10.18	77.00 ± 12.66	0.063 ± 0.006	0.051 ± 0.005	0.081 ± 0.004	0.067 ± 0.009	4/4	3/3
**D5**	14.75 ± 2.72	13.00 ± 0.71	75.75 ± 18.08	60.75 ± 5.31	0.046 ± 0.005	0.059 ± 0.004	0.055 ± 0.004	0.063 ± 0.005	3/4	4/4
P. nodosum										
**W7**	16.25 ± 3.04	19.25 ± 3.57	126.75 ± 20.87	136.00 ± 13.95	0.049 ± 0.001	0.049 ± 0.006	0.074 ± 0.002	0.070 ± 0.006	1/4	3/4
**W8**	28.50 ± 5.52	25.50 ± 1.32	205.25 ± 34.99	162.00 ± 8.80	0.060 ± 0.007	0.063 ± 0.003	0.084 ± 0.006	0.083 ± 0.003	3/4	2/4
**W9**	16.25 ± 2.46	11.00 ± 2.20	170.25 ± 36.48	106.25 ± 15.27	0.067 ± 0.005	0.058 ± 0.008	**0.106 ± 0.010**	**0.091 ± 0.004**	3/4	3/4
**D6**	30.25 ± 6.42	25.25 ± 3.86	**298.75 ± 38.51**	**205.75 ± 30.71**	0.064 ± 0.003	0.059 ± 0.006	0.097 ± 0.002	0.087 ± 0.001	4/4	4/4
**D7**	18.00 ± 2.08	24.00 ± 5.51	164.67 ± 19.78	162.33 ± 39.35	**0.066 ± 0.001**	**0.086 ± 0.009**	0.101 ± 0.006	0.110 ± 0.007	3/3	3/3
P. alpinum										
**W10**	3.50 ± 1.55	1.75 ± 0.63	17.00 ± 5.37	9.00 ± 2.74	0.038 ± 0.011	0.025 ± 0.009	**0.053 ± 0.006**	**0.031 ± 0.006**	0/4	0/4
**W11**	1.50 ± 0.50	2.75 ± 0.75	15.25 ± 0.48	21.75 ± 2.32	0.026 ± 0.011	0.028 ± 0.008	0.043 ± 0.005	0.050 ± 0.005	0/4	0/4
**D8**	2.33 ± 0.88	2.33 ± 0.33	12.00 ± 5.51	8.67 ± 1.45	0.043 ± 0.012	0.029 ± 0.003	0.046 ± 0.012	0.042 ± 0.008	0/3	0/3

Data in bold indicates significant difference between NWL and WL ($p < 0.05$, sub-design ANOVA).

Table S4. Root traits of *P. pratense*, *P. nodosum* and *P. alpinum* in non-waterlogging (NWL) and waterlogging (WL) at the end of the 21 days treatment. **.** Root dry weight (RDW), percent root weight of total plant dry weight (%RDW) and ratio of dry weight of the upper 5cm of the root system to the dry weight of the total root (5RDW:RDW). Values are means and standard error (± SE) of plants within an accession.

		RDW (g)		%RDW		5RDW:RDW	
**Acc. ID-SLU**	**Species**	**NWL**	**WL**	**NWL**	**WL**	**NWL**	**WL**
W1	*P. pratense*	1.22 ± 0.18	1.24 ± 0.13	19.55 ± 1.06	16.60 ± 0.99	**0.38 ± 0.03**	**0.48 ± 0.02**
W2	*P. pratense*	1.77 ± 0.47	1.15 ± 0.14	**21.34 ± 2.74**	**14.35 ± 1.29**	**0.30 ± 0.02**	**0.56 ± 0.03**
W3	*P. pratense*	1.92 ± 0.34	1.37 ± 0.05	**18.71 ± 0.60**	**13.27 ± 0.82**	**0.25 ± 0.00**	**0.45 ± 0.02**
W4	*P. pratense*	**2.68 ± 0.38**	**1.54 ± 0.11**	**27.99 ± 2.43**	**19.27 ± 1.62**	**0.25 ± 0.01**	**0.42 ± 0.03**
W5	*P. pratense*	**2.43 ± 0.08**	**1.44 ± 0.17**	19.40 ± 0.76	15.45 ± 1.64	**0.27 ± 0.01**	**0.44 ± 0.06**
W6	*P. pratense*	**1.71 ± 0.15**	**2.48 ± 0.15**	18.50 ± 2.78	20.43 ± 1.91	**0.28 ± 0.02**	**0.40 ± 0.05**
D1	*P. pratense*	2.14 ± 0.20	2.11 ± 0.21	18.53 ± 0.74	19.23 ± 1.03	**0.29 ± 0.03**	**0.41 ± 0.06**
D2	*P. pratense*	1.91 ± 0.24	1.50 ± 0.22	18.96 ± 1.63	15.67 ± 2.69	**0.29 ± 0.03**	**0.46 ± 0.06**
D3	*P. pratense*	2.59 ± 0.41	2.25 ± 0.20	22.47 ± 1.25	18.23 ± 1.81	0.28 ± 0.02	0.39 ± 0.03
D4	*P. pratense*	1.56 ± 0.15	1.21 ± 0.27	18.62 ± 2.67	15.31 ± 1.30	0.25 ± 0.02	0.34 ± 0.07
D5	*P. pratense*	**2.79 ± 0.31**	**1.09 ± 0.26**	**26.76 ± 3.83**	**13.66 ± 0.50**	**0.30 ± 0.07**	**0.51 ± 0.05**
**Mean ± SE**		2.07 ± 0.11	1.59 ± 0.09	20.98 ± 0.75	16.52 ± 0.55	0.29 ± 0.01	0.45 ± 0.02
W7	*P. nodosum*	2.36 ± 0.46	2.76 ± 0.24	22.27 ± 1.85	20.63 ± 2.55	**0.24 ± 0.02**	**0.45 ± 0.03**
W8	*P. nodosum*	**1.90 ± 0.32**	**0.73 ± 0.10**	**18.73 ± 1.17**	**12.83 ± 1.34**	**0.27 ± 0.02**	**0.70 ± 0.05**
W9	*P. nodosum*	0.55 ± 0.08	0.24 ± 0.05	**14.38 ± 1.53**	**7.90 ± 0.77**	**0.35 ± 0.07**	**0.77 ± 0.05**
D6	*P. nodosum*	1.71 ± 0.43	1.00 ± 0.29	**18.42 ± 2.97**	**11.79 ± 1.23**	**0.34 ± 0.03**	**0.66 ± 0.04**
D7	*P. nodosum*	0.77 ± 0.08	0.41 ± 0.05	17.62 ± 0.27	12.37 ± 1.67	**0.31 ± 0.01**	**0.69 ± 0.06**
**Mean ± SE**		1.43 ± 0.21	1.06 ± 0.23	18.32 ± 0.97	13.14 ± 1.19	0.30 ± 0.02	0.65 ± 0.03
W10	*P. alpinum*	0.53 ± 0.15	0.25 ± 0.02	**29.16 ± 2.51**	**17.73 ± 1.61**	**0.27 ± 0.03**	**0.73 ± 0.04**
W11	*P. alpinum*	**0.63 ± 0.02**	**0.27 ± 0.02**	**54.91 ± 1.37**	**37.33 ± 7.85**	**0.21 ± 0.01**	**0.71 ± 0.04**
D8	*P. alpinum*	0.51 ± 0.21	0.21 ± 0.10	**34.72 ± 7.37**	**20.53 ± 3.25**	**0.40 ± 0.19**	**0.68 ± 0.03**
**Mean ± SE**		0.56 ± 0.07	0.25 ± 0.03	40.04 ± 4.12	25.62 ± 3.94	0.28 ± 0.05	0.71 ± 0.02

Data in bold indicates significant difference between NWL and WL ($p < 0.05$, ANOVA)

Table S5. Root anatomy traits of accessions of *Phleum pratense*, *P. nodosum* and *P. alpinum* in non-waterlogging (NWL) and waterlogging (WL) conditions at the end of the 21 days treatment. The traits shown are root cross section area (RA), stele area (SA), ratios of the cortex area to root cross section area (CEA:RA), the aerenchyma to cortex area (AA:CEA), the cortex to stele area (CEA:SA) and the stele area to root cross section area (SA:RA). Values are means and standard error (± SE) of plants within an accession.

		RA (mm^2)		SA (mm^2)		CEA:RA		AA:CEA		CEA:SA		SA:RA	
**Acc. ID-SLU**	**Species**	**NWL**	**WL**	**NWL**	**WL**	**NWL**	**WL**	**NWL**	**WL**	**NWL**	**WL**	**NWL**	**WL**
W1	*P. pratense*	1.47 ± 0.19	1.82 ± 0.15	0.17 ± 0.03	0.16 ± 0.01	**0.89 ± 0.01**	**0.91 ± 0.01**	**0.026 ± 0.010**	**0.27 ± 0.03**	**8.0±0.6**	**10.9±0.9**	**0.11 ± 0.01**	**0.09 ± 0.01**
W2	*P. pratense*	1.64 ± 0.14	1.92 ± 0.25	0.20 ± 0.02	0.19 ± 0.04	**0.88 ± 0.01**	**0.90 ± 0.01**	**0.046 ± 0.012**	**0.26 ± 0.01**	**7.2±0.5**	**9.9±1.2**	**0.13 ± 0.01**	**0.10 ± 0.01**
W3	*P. pratense*	1.69 ± 0.20	2.07 ± 0.24	0.23 ± 0.03	0.19 ± 0.02	**0.87 ± 0.01**	**0.91 ± 0.01**	**0.039 ± 0.017**	**0.34 ± 0.02**	**6.7±0.5**	**10±0.8**	**0.14 ± 0.01**	**0.10 ± 0.01**
W4	*P. pratense*	1.60 ± 0.11	1.78 ± 0.11	0.20 ± 0.03	0.18 ± 0.02	**0.88 ± 0.01**	**0.90 ± 0.01**	**0.031 ± 0.015**	**0.28 ± 0.02**	**7.4±0.5**	**9.5±0.7**	**0.12 ± 0.01**	**0.10 ± 0.01**
W5	*P. pratense*	**1.78 ± 0.12**	**2.45 ± 0.17**	0.24 ± 0.02	0.29 ± 0.01	0.86 ± 0.01	0.88 ± 0.01	**0.060 ± 0.013**	**0.33 ± 0.03**	6.5±0.4	7.5±0.4	0.14 ± 0.01	0.12 ± 0.01
W6	*P. pratense*	**1.32 ± 0.14**	**2.26 ± 0.19**	**0.15 ± 0.02**	**0.24 ± 0.02**	0.88 ± 0.01	0.89 ± 0.00	**0.032 ± 0.016**	**0.34 ± 0.04**	7.7±0.8	8.4±0.3	0.12 ± 0.01	0.11 ± 0.00
D1	*P. pratense*	1.65 ± 0.11	1.31 ± 0.45	**0.29 ± 0.02**	**0.13 ± 0.04**	**0.82 ± 0.01**	**0.90 ± 0.01**	**0.022 ± 0.009**	**0.32 ± 0.03**	**4.8±0.3**	**9.1±0.9**	**0.18 ± 0.01**	**0.11 ± 0.01**
D2	*P. pratense*	1.59 ± 0.15	1.54 ± 0.19	0.27 ± 0.03	0.22 ± 0.03	**0.83 ± 0.01**	**0.86 ± 0.00**	**0.062 ± 0.020**	**0.21 ± 0.03**	5.0±0.4	6.0±0.2	**0.17 ± 0.01**	**0.14 ± 0.00**
D3	*P. pratense*	**1.99 ± 0.20**	**2.96 ± 0.24**	0.27 ± 0.02	0.26 ± 0.02	**0.86 ± 0.01**	**0.91 ± 0.01**	**0.069 ± 0.020**	**0.36 ± 0.03**	**6.4±0.5**	**10.7±1.2**	**0.14 ± 0.01**	**0.09 ± 0.01**
D4	*P. pratense*	**1.64 ± 0.13**	**2.20 ± 0.16**	0.23 ± 0.02	0.25 ± 0.03	**0.86 ± 0.01**	**0.89 ± 0.01**	**0.063 ± 0.017**	**0.31 ± 0.03**	**6.3±0.3**	**8.2±0.9**	**0.14 ± 0.01**	**0.12 ± 0.01**
D5	*P. pratense*	2.03 ± 0.20	1.78 ± 0.17	0.30 ± 0.04	0.25 ± 0.03	0.85 ± 0.01	0.86 ± 0.01	0.081 ± 0.029	0.11 ± 0.03	5.8±0.5	6.4±0.4	0.15 ± 0.01	0.14 ± 0.01
**Mean ± SE**		1.66 ± 0.05	2.03 ± 0.08	0.23 ± 0.009	0.21 ± 0.009	0.861 ± 0.003	0.892 ± 0.003	0.047 ± 0.005	0.288 ± 0.011	6.5±0.2	8.8±0.3	0.139 ± 0.003	0.108 ± 0.003
W7	*P.nodosum*	**2.05 ± 0.11**	**2.59 ± 0.15**	**0.32 ± 0.02**	**0.26 ± 0.02**	**0.85 ± 0.00**	**0.90 ± 0.01**	**0.065 ± 0.026**	**0.45 ± 0.02**	**5.5±0.2**	**9.3±0.7**	**0.16 ± 0.00**	**0.10 ± 0.01**
W8	*P.nodosum*	0.92 ± 0.06	1.13 ± 0.11	0.15 ± 0.01	0.13 ± 0.01	**0.84 ± 0.01**	**0.88 ± 0.01**	**0.000 ± 0.000**	**0.20 ± 0.03**	**5.6±0.6**	**7.6±0.6**	**0.16 ± 0.01**	**0.12 ± 0.01**
W9	*P.nodosum*	0.59 ± 0.06	0.79 ± 0.05	0.09 ± 0.01	0.09 ± 0.01	**0.86 ± 0.01**	**0.89 ± 0.01**	**0.000 ± 0.000**	**0.14 ± 0.02**	**6.0±0.3**	**8.4±0.6**	**0.14 ± 0.01**	**0.11 ± 0.01**
D6	*P.nodosum*	**0.78 ± 0.12**	**1.22 ± 0.10**	**0.13 ± 0.02**	**0.17 ± 0.02**	**0.84 ± 0.01**	**0.86 ± 0.01**	**0.008 ± 0.008**	**0.18 ± 0.04**	5.2±0.4	6.3±0.3	**0.17 ± 0.01**	**0.14 ± 0.01**
D7	*P.nodosum*	**0.88 ± 0.08**	**1.34 ± 0.25**	0.12 ± 0.01	0.15 ± 0.02	**0.86 ± 0.01**	**0.89 ± 0.01**	**0.000 ± 0.000**	**0.17 ± 0.03**	**6.4±0.3**	**7.9±0.5**	**0.14 ± 0.01**	**0.11 ± 0.01**
**Mean ± SE**		1.05 ± 0.09	1.38 ± 0.12	0.16 ± 0.015	0.16 ± 0.011	0.848 ± 0.004	0.883 ± 0.004	0.015 ± 0.007	0.221 ± 0.021	5.2±0.2	6.1±0.3	0.152 ± 0.004	0.117 ± 0.004
W10	*P. alpinum*	**1.38 ± 0.12**	**1.77 ± 0.08**	0.26 ± 0.02	0.27 ± 0.03	**0.81 ± 0.01**	**0.85 ± 0.02**	**0.037 ± 0.014**	**0.15 ± 0.04**	4.3±0.3	5.8±0.8	**0.19 ± 0.01**	**0.15 ± 0.02**
W11	*P. alpinum*	1.38 ± 0.09	1.51 ± 0.08	0.21 ± 0.02	0.22 ± 0.01	0.85 ± 0.01	0.85 ± 0.01	**0.037 ± 0.015**	**0.12 ± 0.03**	5.7±0.3	5.9±0.4	0.15 ± 0.01	0.15 ± 0.01
D8	*P. alpinum*	**1.19 ± 0.16**	**1.63 ± 0.14**	0.18 ± 0.02	0.22 ± 0.02	0.85 ± 0.01	0.86 ± 0.01	**0.020 ± 0.013**	**0.13 ± 0.02**	5.5±0.2	6.5±0.6	0.15 ± 0.01	0.14 ± 0.01
**Mean ± SE**		1.33 ± 0.07	1.61 ± 0.07	0.22 ± 0.013	0.23 ± 0.011	0.836 ± 0.006	0.854 ± 0.006	0.033 ± 0.008	0.131 ± 0.015	5.7±0.2	7.9±0.3	0.165 ± 0.006	0.146 ± 0.006

Data in bold indicates significant difference between NWL and WL ($p < 0.05$, ANOVA).

Table S6. WL response index of *P. pratense*, *P. nodosum* and *P. alpinum*, calculated as the proportional difference between the WL and NWL.

Traits	*P. pratense*	*P. nodosum*	*P. alpinum*
SDW	0.034^{a} ± 0,043	-0.014^{a} ± 0,093	-0.025^{a} ± 0,135
RGR-LN	0.016^{a} ± 0,028	-0.048^{a} ± 0,029	-0.106^{a} ± 0,103
RGR-TN	-0.034^{a} ± 0,037	0.013^{a} ± 0,053	0.028^{a} ± 0,144
%RDW	-0.198^{a} ± 0,032	-0.320^{ab} ± 0,045	-0.370^{b} ± 0,056
5RDW:RDW	0.573^{a} ± 0,056	1.062^{b} ± 0,093	1.717^{c} ± 0,259
AA:CEA	6.337^{b} ± 0,436	15.62^{a*} ± 2,678	3.77^{b} ± 0,605
CEA:SA	0.358^{a} ± 0,043	0.374^{a} ± 0,049	0.152^{a} ± 0,062

*Based on two accession that formed AA in NWL conditions. Three accessions have no AA in NWL.
